# New universal ITS2 primers for high-resolution herbivory analyses using DNA metabarcoding in both tropical and temperate zones

**DOI:** 10.1038/s41598-018-26648-2

**Published:** 2018-06-04

**Authors:** Rosemary J. Moorhouse-Gann, Jenny C. Dunn, Natasha de Vere, Martine Goder, Nik Cole, Helen Hipperson, William O. C. Symondson

**Affiliations:** 1 School of Biosciences, Cardiff University, The Sir Martin Evans Building, Museum Avenue, Cardiff, CF10 3AX UK; 2 RSPB Centre for Conservation Science, Royal Society for the Protection of Birds, The Lodge, Potton Road, Sandy, Bedfordshire SG19 2DL UK; 3 School of Life Sciences, University of Lincoln, Joseph Banks Laboratories, Green Lane, Lincoln, LN6 7TS UK; 4 National Botanic Garden of Wales, Llanarthne, Carmarthenshire SA32 8HG UK; 5 Institute of Biological, Environmental & Rural Sciences (IBERS), Aberystwyth University, Penglais, Aberystwyth, Ceredigion SY23 3DA UK; 6 Mauritian Wildlife Foundation, Grannum Road, Vacoas, Mauritius; 7 Durrell Wildlife Conservation Trust, Les Augrès Manor, La Profonde Rue, Trinity, JE3 5BP Jersey Channel Islands, UK; 8 NERC Biomolecular Analysis Facility, Department of Animal and Plant Sciences, University of Sheffield, Western Bank, Sheffield, S10 2TN UK

## Abstract

DNA metabarcoding is a rapidly growing technique for obtaining detailed dietary information. Current metabarcoding methods for herbivory, using a single locus, can lack taxonomic resolution for some applications. We present novel primers for the second internal transcribed spacer of nuclear ribosomal DNA (ITS2) designed for dietary studies in Mauritius and the UK, which have the potential to give unrivalled taxonomic coverage and resolution from a short-amplicon barcode. *In silico* testing used three databases of plant ITS2 sequences from UK and Mauritian floras (native and introduced) totalling 6561 sequences from 1790 species across 174 families. Our primers were well-matched *in silico* to 88% of species, providing taxonomic resolution of 86.1%, 99.4% and 99.9% at the species, genus and family levels, respectively. *In vitro*, the primers amplified 99% of Mauritian (n = 169) and 100% of UK (n = 33) species, and co-amplified multiple plant species from degraded faecal DNA from reptiles and birds in two case studies. For the ITS2 region, we advocate taxonomic assignment based on best sequence match instead of a clustering approach. With short amplicons of 187–387 bp, these primers are suitable for metabarcoding plant DNA from faecal samples, across a broad geographic range, whilst delivering unparalleled taxonomic resolution.

## Introduction

Analysis of trophic interactions facilitates our understanding of community ecology and ecosystem functioning. Analysing such complex and dynamic processes can benefit conservation by informing management strategies. For example, monitoring dietary composition allows for human-wildlife conflict to be detected and monitored^1^, for the costs^2^ and potential benefits^3^ of alien species to be assessed, for understanding how habitat management influences food webs^4^, and for understanding seed dispersal and pollination networks to inform ecosystem restoration^5–8^. An understanding of trophic links also allows species at risk due to inflexible niches to be identified, isolates particularly vulnerable interaction networks, and allows for suitable (re)introduction sites to be identified^9–11^. Large herbivores in particular are recognised as keystone consumers^1,12^ and determining their diets can be critical to understanding their impact on plant communities and the wider food web. This is particularly relevant in the light of recent rewilding efforts, including the introduction of non-native species as ecological replacements (analogues) for extinct taxa to restore ecosystem function, or the conservation or reintroduction of native species^1,12^.

Traditional methods of dietary analysis, such as the morphological examination of faecal samples and gut contents, or feeding observations, are fraught with methodological problems. Molecular methods provide an alternative suite of approaches that can generate greater volumes of data more rapidly and with greater precision^13^, and comparisons between morphological and molecular methods show that molecular analysis generally provide greater sensitivity^3,14^. Species-specific primers can be used to detect the DNA of particular focal dietary items in gut contents or faecal samples^15–17^. However, this approach is only appropriate if *a priori* dietary information is available and if the dietary range is small. It cannot unravel the effects that non-focal species may be having on dietary selection by a highly polyphagous predator or herbivore. In order to overcome such problems, and to determine whole dietary ranges, DNA barcodes coupled with next generation sequencing (NGS), often referred to as DNA metabarcoding, have been widely adopted.

A key target for designing metabarcoding primers is to maximise the taxonomic coverage of a primer set to ensure all potential target species are amplified. However, this often leads to reduced taxonomic resolution, as the highly conserved primer sites required for maximising coverage often favour less variable DNA regions, resulting in reduced ability to distinguish between taxa^18^. Thus, the panacea for metabarcoding is primers with high taxonomic coverage that amplify a gene region with high taxonomic resolution. An additional challenge for dietary analyses is for this gene region to be short enough to be reliably amplified from degraded samples.

Identification of animal dietary components primarily uses the mitochondrial cytochrome *c* oxidase gene, which has been shown to effectively resolve species identity^19–21^. However, in plants the mitochondrial genome evolves too slowly for these genes to provide sufficient variation to be useful barcodes^22^. In 2009, the Consortium for the Barcode of Life approved plastid *matK* and *rbcL* as the barcode regions for use in land plants^23^. Unfortunately, the large fragment size (*rbcL* = 654 bp; *matK* = 889 bp)^24^ of these barcodes makes them impractical for dietary metabarcoding studies. Minibarcodes have been designed within *rbcL*, but those suitable for application in dietary studies have low discriminatory power at the species level^25^. The most commonly used DNA barcode in herbivory studies is the P6 loop of the plastid *trn*L (UAA) gene^1,3,14,22,26–31^, but *in silico* analysis of this barcoding region using the EMBL database^32^ estimated taxonomic resolution to be around 18% at the species level^18^. Whilst *in vitro* studies using this region report species level taxonomic assignment of 29.8%^33^ to 77%^34^, there remains room for improvement. The second internal transcribed spacer (ITS2) of nuclear ribosomal DNA has been suggested as a ‘gold standard’ barcode for identifying plants^35^ and there is growing evidence to support this^36,37^. In a study examining 4800 species of medicinal plants, testing the most variable region of a larger ITS2 amplicon as a barcoding region, correct taxonomic identification at the species and genus levels was approximately 91.5% and 99.8%^35^. Such high taxonomic resolution mostly confined to a 160–320 bp region makes ITS2 a promising DNA barcoding region for use in dietary studies.

General primers for ITS2 have been designed for priming sites within the more conserved flanking regions of 5.8S and 26S^35,38^. This presents a problem for dietary studies since the resultant amplicon length (approximately 387–547 bp using S2F and S3R^35^) is potentially too great to be reliably detected in semi-digested samples. Designing shorter amplicon primers closer to ITS2 within the flanking regions, or within ITS2 itself, is a challenge due to the high interspecific variation that has the potential to provide such high taxonomic resolution^35^ but could limit taxonomic coverage. Additionally, ITS2 presents challenges in interpretation due to the presence of paralogous gene copies and the potential for co-amplification of non-target fungal amplicons^36^.

Here, we describe primers initially designed for two in-depth dietary studies: a suite of Mauritian herbivores^39^, and UK doves and pigeons^40^. We test the scope of these primers for wider herbivory studies by running analyses against three ITS2 sequence databases: (1) a comprehensive database of plants from two Mauritian islands (Mauritian database); (2) all species known to feature in the diet of an obligate granivore (European turtle dove *Streptopelia turtur*; UK columbid database); and (3) a database consisting of UK plant sequences downloaded from GenBank (UK database). This last database consists largely of vouchered specimens and, where available, contains at least one representative species from each genus of plant present in the UK.

We used these databases to address three objectives:

1. To establish the taxonomic coverage of our new primers, against all three databases *in silico* and against all available Mauritian species and a subset of UK species *in vitro*.
2. To determine the taxonomic resolution of our primers using all three databases combined for the ITS2 region.
3. For the two databases with multiple sequences per species (Mauritian and a subset of the UK database), identify clustering thresholds to use in the bioinformatics pipeline for analysis of NGS data, to maximise taxonomic resolution and minimise assignment of multiple haplotypes of the same species to different molecular operational taxonomic units (MOTUs).

To confirm that our primers successfully co-amplify a diverse range of plant species within the same degraded faecal samples, from both birds and reptiles, we also present detailed dietary data from an omnivorous reptile species (Mauritius: Telfair’s skink *Leiolopisma telfairii*) and an herbivorous bird species (UK: stock dove *Columba oenas*).

## Results

### *In silico* testing of primers

**Figure 1:**
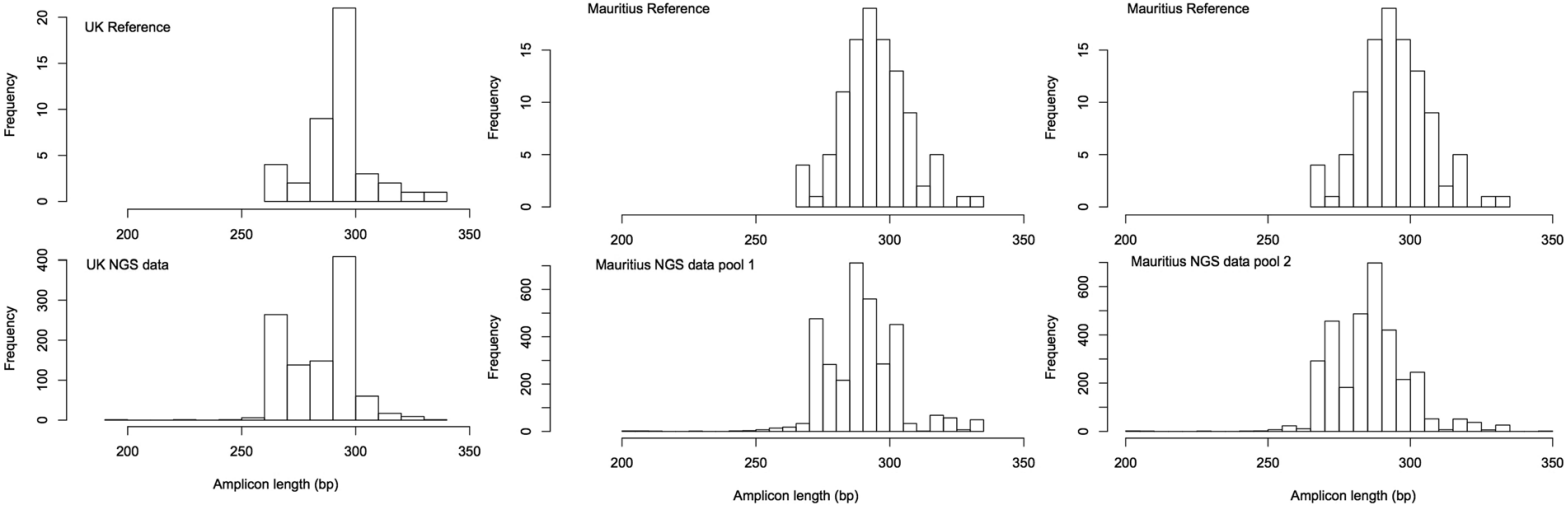
Comparison of amplicon length distribution from available species and NGS datasets for (**a**) UK dove and pigeon diet, (**b**) Telfair’s skink diet pool 1 and (**c**) Telfair’s skink diet pool 2.

Across all three databases, amplicon lengths, minus priming sites, ranged from 187–387 bp (Table 1; Fig. 1). Where coverage of both forward and reverse primer binding regions was available, 88% of Mauritian (n = 131 species, 114 genera, 57 families; Table 2) and 89% of UK plants (n = 986 species, 561 genera and 121 families; Table 3) fulfilled the primer fit criteria (with fewer than 3 bp mismatches and no mismatch within the last 2 bp at the 3′ end). Poor primer matches (where 50% or fewer of tested species fulfilled the primer fit criteria) were found in only 3 families within the UK (Hydrocharitaceae = 50%, n = 6; Cyperaceae = 0%, n = 44, Thymelaeaceae = 50%, n = 2) where multiple species were tested (Table 3). In the Mauritian database, *in silico* primer fit was particularly poor for Cyperaceae (0%, n = 4) and Moraceae (50%, n = 2). Analyses of matches for forward and reverse primers independently, due to short sequence lengths, found particularly poor fit for Cyperaceae in both databases due to poor reverse primer fit (0%, Mauritius n = 3; UK n = 79), and Orchidaceae in Mauritius (0%, n = 2) but not in the UK (see Supplementary Table S1a for the Mauritian database, and Supplementary Tables S1b,c for the UK databases).

**Table 1:** Mean amplicon lengths among families from sequences in our combined database.

Order	Family	No. species	Mean ± SE amplicon length (bp)
Lamiales	Acanthaceae	2	291 ± 7.49
Sapindales	Aceraceae	1	310 ± 0
Acorales	Acoraceae	1	330 ± 0
Dipsacales	Adoxaceae	3	298 ± 0.88
Caryophyllales	Aizoaceae	1	275 ± 0
Alismatales	Alismataceae	6	365 ± 8.87
Caryophyllales	Amaranthaceae	9	293 ± 4.22
Asparagales	Amaryllidaceae	6	311 ± 1.83
Sapindales	Anacardiaceae	2	297 ± 3.25
Apiales	Apiaceae	31	300 ± 0.75
Gentianales	Apocynaceae	3	312 ± 4.18
Alismatales	Aponogetonaceae	1	343 ± 0
Aquifoliales	Aquifoliaceae	1	307 ± 0
Alismatales	Araceae	4	334 ± 17.2
Apiales	Araliaceae	4	301 ± 0.86
Pinales	Araucariaceae	1	319 ± 0
Asparagales	Asparagaceae	5	300 ± 18.7
Asterales	Asteraceae	95	295 ± 0.73
Ericales	Balsaminaceae	1	268 ± 0
Ranunculales	Berberidaceae	1	294 ± 0
Fagales	Betulaceae	6	301 ± 0.94
Lamiales	Bignoniaceae	1	310 ± 0
Boraginales	Boraginaceae	17	298 ± 0.81
Brassicales	Brassicaceae	53	264 ± 0.33
Alismatales	Butomaceae	1	346 ± 0
Buxales	Buxaceae	1	305 ± 0
Nymphaeales	Cabombaceae	1	279 ± 0
Lamiales	Calceolariaceae	1	300 ± 0
Asterales	Campanulaceae	10	330 ± 6.52
Rosales	Cannabaceae	2	298 ± 6.5
Dipsacales	Caprifoliaceae	5	302 ± 2.24
Brassicales	Caricaceae	1	305 ± 0
Caryophyllales	Caryophyllaceae	47	294 ± 1.27
Celastrales	Celastraceae	3	293 ± 3.52
Ceratophyllales	Ceratophyllaceae	2	329 ± 0
Caryophyllales	Chenopodiaceae	13	302 ± 0.63
Malvales	Cistaceae	1	280 ± 0
Myrtales	Combretaceae	1	284 ± 0
Commelinales	Commelinaceae	1	301 ± 0
Solanales	Convolvulaceae	8	287 ± 3.93
Saxifragales	Crassulaceae	5	307 ± 6.55
Cucurbitales	Cucurbitaceae	3	320 ± 3.87
Pinales	Cupressaceae	3	292 ± 2.02
Ericales	Diapensiaceae	1	300 ± 0
Caryophyllales	Droseraceae	2	307 ± 4
Ericales	Ebenaceae	1	318 ± 0
Ericales	Ericaceae	15	305 ± 1.38
Malpighiales	Erythroxylaceae	1	295 ± 0
Malpighiales	Euphorbiaceae	15	289 ± 2.67
Fabales	Fabaceae	61	292 ± 0.78
Fagales	Fagaceae	2	286 ± 0
Gentianales	Gentianaceae	7	306 ± 0.89
Geraniales	Geraniaceae	13	310 ± 0.55
Asterales	Goodeniaceae	1	310 ± 0
Apiales	Griseliniaceae	1	306 ± 0
Gunnerales	Gunneraceae	1	296 ± 0
Saxifragales	Haloragaceae	1	292 ± 0
Boraginales	Heliotropiaceae	1	292 ± 0
Asparagales	Hyacinthaceae	2	292 ± 3.5
Cornales	Hydrangeaceae	1	302 ± 0
Alismatales	Hydrocharitaceae	3	274 ± 11.1
Boraginales	Hydrophyllaceae	1	294 ± 0
Malpighiales	Hypericaceae	7	310 ± 0.71
Asparagales	Iridaceae	2	307 ± 2.5
Fagales	Juglandaceae	1	294 ± 0
Poales	Juncaceae	23	303 ± 1.31
Alismatales	Juncaginaceae	1	324 ± 0
Lamiales	Lamiaceae	15	300 ± 2.36
Laurales	Lauraceae	2	299 ± 10.5
Lamiales	Lentibulariaceae	3	320 ± 10.7
Liliales	Liliaceae	4	298 ± 8.18
Malpighiales	Linaceae	1	298 ± 0
Myrtales	Lythraceae	2	295 ± 1
Malvales	Malvaceae	16	303 ± 1.90
Liliales	Melanthiaceae	1	303 ± 0
Sapindales	Meliaceae	1	307 ± 0
Asterales	Menyanthaceae	2	307 ± 10.5
Caryophyllales	Montiaceae	2	287 ± 1.5
Rosales	Moraceae	2	316 ± 9.24
Fagales	Myricaceae	1	301 ± 0
Myrtales	Myrtaceae	3	285 ± 2
Caryophyllales	Nyctaginaceae	1	283 ± 0
Nymphaeales	Nymphaeaceae	1	327 ± 0
Lamiales	Oleaceae	4	293 ± 2.17
Myrtales	Onagraceae	10	291 ± 1.38
Asparagales	Orchidaceae	15	321 ± 2.18
Lamiales	Orobanchaceae	24	303 ± 1.47
Oxalidales	Oxalidaceae	2	301 ± 1.25
Ranunculales	Papaveraceae	9	311 ± 5.76
Malpighiales	Passifloraceae	1	269 ± 0
Caryophyllales	Petiveriaceae	1	292 ± 0
Malpighiales	Phyllanthaceae	3	279 ± 3.12
Caryophyllales	Phytolaccaceae	1	296 ± 0
Pinales	Pinaceae	3	312 ± 4.91
Apiales	Pittosporaceae	1	305 ± 0
Lamiales	Plantaginaceae	24	288 ± 1.34
Proteales	Platanaceae	1	311 ± 0
Caryophyllales	Plumbaginaceae	2	321 ± 4.25
Poales	Poaceae	96	291 ± 0.35
Fabales	Polygalaceae	2	294 ± 1
Caryophyllales	Polygonaceae	10	286 ± 7.09
Caryophyllales	Portulacaceae	1	292 ± 0
Alismatales	Potamogetonaceae	6	337 ± 6.12
Ericales	Primulaceae	6	287 ± 2.84
Polypodiales	Pteridaceae	1	253 ± 0
Ranunculales	Ranunculaceae	18	287 ± 1.22
Brassicales	Resedaceae	1	289 ± 0
Rosales	Rhamnaceae	4	288 ± 3.70
Rosales	Rosaceae	61	287 ± 0.54
Gentianales	Rubiaceae	8	297 ± 6.53
Sapindales	Rutaceae	1	307 ± 0
Malpighiales	Salicaceae	16	289 ± 0.49
Santalales	Santalaceae	1	293 ± 0
Sapindales	Sapindaceae	1	300 ± 0
Ericales	Sapotaceae	1	309 ± 0
Saxifragales	Saxifragaceae	13	311 ± 1.08
Lamiales	Scrophulariaceae	6	299 ± 1.40
Selaginellales	Selaginellaceae	1	233 ± 0
Sapindales	Simaroubaceae	1	296 ± 0
Solanales	Solanaceae	11	287 ± 2.78
Caryophyllales	Tamaricaceae	1	319 ± 0
Pinales	Taxaceae	1	303 ± 0
Santalales	Thesiaceae	1	289 ± 0
Malvales	Thymelaeaceae	2	292 ± 0.74
Alismatales	Tofieldiaceae	1	307 ± 0
Poales	Typhaceae	4	296 ± 8.79
Rosales	Ulmaceae	2	290 ± 1
Rosales	Urticaceae	3	311 ± 5.08
Lamiales	Verbenaceae	2	303 ± 1.5
Malpighiales	Violaceae	8	284 ± 0.61
Vitales	Vitaceae	1	331 ± 0
Asparagales	Xanthorrhoeaceae	1	312 ± 0
Alismatales	Zosteraceae	1	310 ± 0

**Table 2:** Results of *in silico* and *in vitro* analysis of primer fit for UniPlantF and UniPlantR for Mauritian plants at the species level, summarised by family.

Order	Family	Tested *in silico*	*In silico* matches	% matches	Tested *in vitro*	Amplified *in vitro*	% Amplified
Apiales	Araliaceae	1	1	100	1	1	100
Arecales	Arecaceae				3	3	100
Asparagales	Amaryllidaceae	1	1	100	1	1	100
Asparagales	Asparagaceae	3	3	100	3	3	100
Asparagales	Orchidaceae	1	0	0	3	3	100
Asparagales	Xanthorrhoeaceae				1	1	100
Asterales	Asteraceae	7	7	100	8	8	100
Asterales	Campanulaceae	1	1	100	1	1	100
Asterales	Goodeniaceae	1	1	100	1	1	100
Boraginales	Boraginaceae	1	1	100	3	3	100
Brassicales	Caricaceae	1	1	100	1	1	100
Caryophyllales	Aizoaceae				1	1	100
Caryophyllales	Amaranthaceae	4	4	100	4	4	100
Caryophyllales	Nyctaginaceae	1	1	100	1	1	100
Caryophyllales	Petiveriaceae	1	1	100	1	1	100
Caryophyllales	Portulacaceae	1	1	100	1	1	100
Celastrales	Celastraceae	2	2	100	2	2	100
Commelinales	Commelinaceae	1	1	100	1	1	100
Ericales	Ebenaceae	1	1	100	3	3	100
Ericales	Lecythidaceae				1	1	100
Ericales	Sapotaceae	1	1	100	1	1	100
Fabales	Fabaceae	13	11	85	13	13	100
Gentianales	Apocynaceae	4	4	100	6	6	100
Gentianales	Rubiaceae	5	5	100	5	5	100
Lamiales	Acanthaceae	1	1	100	2	2	100
Lamiales	Bignoniaceae	1	1	100	1	1	100
Lamiales	Lamiaceae	1	1	100	1	1	100
Lamiales	Oleaceae	1	1	100	2	2	100
Lamiales	Scrophulariaceae	1	1	100	1	1	100
Lamiales	Verbenaceae	1	1	100	2	2	100
Laurales	Lauraceae	1	1	100	3	3	100
Malpighiales	Erythroxylaceae	1	1	100	1	1	100
Malpighiales	Euphorbiaceae	8	8	100	8	8	100
Malpighiales	Passifloraceae	2	2	100	2	2	100
Malpighiales	Phyllanthaceae	4	4	100	7	7	100
Malpighiales	Salicaceae	2	2	100	3	3	100
Malvales	Malvaceae	7	7	100	8	8	100
Malvales	Thymelaeaceae	1	1	100	1	1	100
Myrtales	Combretaceae	1	1	100	1	1	100
Myrtales	Lythraceae	1	1	100	1	1	100
Myrtales	Myrtaceae	1	1	100	1	1	100
Oxalidales	Oxalidaceae	1	1	100	1	1	100
Pandanales	Pandanaceae	1	1	100	1	1	100
Poales	Cyperaceae	4	0	0	4	4	100
Poales	Poaceae	12	11	92	16	16	100
Polypodiales	Lomariopsidaceae				1	0	0
Polypodiales	Polypodiaceae	1	0	0	1	1	100
Polypodiales	Pteridaceae	1	0	0	2	2	100
Polypodiales	Thelypteridaceae				1	0	0
Pottiales	Pottiaceae	1	0	0	1	1	100
Psilotales	Psilotaceae	1	0	0	1	1	100
Ranunculales	Papaveraceae	1	0	0	1	1	100
Rosales	Moraceae	2	1	50	3	3	100
Rosales	Rhamnaceae	3	3	100	4	4	100
Santalales	Santalaceae	1	1	100	1	1	100
Sapindales	Anacardiaceae	2	2	100	2	2	100
Sapindales	Burseraceae				1	1	100
Sapindales	Meliaceae	1	1	100	1	1	100
Sapindales	Rutaceae	1	1	100	2	2	100
Sapindales	Sapindaceae	2	2	100	3	3	100
Saxifragales	Crassulaceae	1	1	100	1	1	100
Selaginellales	Selaginellaceae				1	1	100
Solanales	Convolvulaceae	3	3	100	4	4	100
Solanales	Solanaceae	5	3	60	4	4	100
Vitales	Vitaceae	1	1	100	1	1	100
	**Total**	**131**	**115**	**88**	**169**	**167**	**99**

For *in silico* results, matches are where primers fit with a maximum of 3 bp mismatches and no mismatches in the last two bp at the 3 prime end. Data presented here are from sequences where both primer binding sites were available for analysis; details of species tested for either forward or reverse primer matches are given in Supplementary Table S1a.

**Table 3:** Results of *in silico* analysis of primer matching for UniPlantF and UniPlantR for plant families within the two UK databases, at the species level.

Order	Family	UK database		Turtle Dove database		Overall		
		No. tested	No. matches	No. tested	No. matches	No. tested	No. matches	% match
Acorales	Acoraceae	1	1			1	1	100
Alismatales	Alismataceae	6	6			6	6	100
Alismatales	Aponogetonaceae	1	1			1	1	100
Alismatales	Araceae	4	4			4	4	100
Alismatales	Butomaceae	1	1			1	1	100
Alismatales	Cymodoceaceae	1	0			1	0	0
Alismatales	Hydrocharitaceae	6	3			6	3	50
Alismatales	Juncaginaceae	1	1			1	1	100
Alismatales	Potamogetonaceae	6	6			6	6	100
Alismatales	Tofieldiaceae	1	1			1	1	100
Alismatales	Zosteraceae	1	1			1	1	100
Apiales	Apiaceae	34	31	1	1	34	31	91
Apiales	Araliaceae	3	3			3	3	100
Apiales	Griseliniaceae	1	1			1	1	100
Apiales	Pittosporaceae	1	1			1	1	100
Aquifoliales	Aquifoliaceae	1	1			1	1	100
Asparagales	Amaryllidaceae	6	5			6	5	83
Asparagales	Asparagaceae	3	2			3	2	67
Asparagales	Hyacinthaceae	2	2			2	2	100
Asparagales	Iridaceae	2	2			2	2	100
Asparagales	Orchidaceae	19	15			19	15	79
Asparagales	Xanthorrhoeaceae	1	1			1	1	100
Asterales	Asteraceae	92	90	6	6	92	90	98
Asterales	Campanulaceae	9	9			9	9	100
Asterales	Menyanthaceae	2	2			2	2	100
Boraginales	Boraginaceae	17	17			17	17	100
Boraginales	Hydrophyllaceae	1	1			1	1	100
Brassicales	Brassicaceae	59	52	3	3	60	52	87
Brassicales	Resedaceae	1	1			1	1	100
Buxales	Buxaceae	1	1			1	1	100
Caryophyllales	Aizoaceae	1	1			1	1	100
Caryophyllales	Amaranthaceae	5	5			5	5	100
Caryophyllales	Caryophyllaceae	49	46	6	6	50	47	94
Caryophyllales	Chenopodiaceae	12	12	1	1	13	13	100
Caryophyllales	Droseraceae	2	2			2	2	100
Caryophyllales	Montiaceae	2	2			2	2	100
Caryophyllales	Phytolaccaceae	1	1			1	1	100
Caryophyllales	Plumbaginaceae	2	2			2	2	100
Caryophyllales	Polygonaceae	11	10	2	2	11	10	91
Caryophyllales	Portulacaceae	1	1			1	1	100
Caryophyllales	Tamaricaceae	1	1			1	1	100
Celastrales	Celastraceae	1	1			1	1	100
Ceratophyllales	Ceratophyllaceae	2	2			2	2	100
Cornales	Hydrangeaceae	1	1			1	1	100
Cucurbitales	Cucurbitaceae	3	3			3	3	100
Dipsacales	Adoxaceae	3	3			3	3	100
Dipsacales	Caprifoliaceae	5	5			5	5	100
Ericales	Balsaminaceae	1	1			1	1	100
Ericales	Diapensiaceae	1	1			1	1	100
Ericales	Ericaceae	16	15			17	15	88
Ericales	Primulaceae	6	6	1	1	6	6	100
Fabales	Fabaceae	52	49	5	5	55	52	95
Fabales	Polygalaceae	2	2			2	2	100
Fagales	Betulaceae	6	6			6	6	100
Fagales	Fagaceae	2	2			2	2	100
Fagales	Juglandaceae	1	1			1	1	100
Fagales	Myricaceae	1	1			1	1	100
Gentianales	Gentianaceae	7	7			7	7	100
Gentianales	Rubiaceae	4	4	1	1	4	4	100
Geraniales	Geraniaceae	13	13	1	1	13	13	100
Gunnerales	Gunneraceae	1	1			1	1	100
Lamiales	Acanthaceae	1	1			1	1	100
Lamiales	Calceolariaceae	1	1			1	1	100
Lamiales	Gesneriaceae	1	0			1	0	0
Lamiales	Lamiaceae	15	14			15	14	93
Lamiales	Lentibulariaceae	4	3			4	3	75
Lamiales	Oleaceae	3	3			3	3	100
Lamiales	Orobanchaceae	24	24			24	24	100
Lamiales	Plantaginaceae	23	22	2	2	25	24	96
Lamiales	Scrophulariaceae	5	5			5	5	100
Lamiales	Verbenaceae	1	1			1	1	100
Liliales	Liliaceae	5	4			5	4	80
Liliales	Melanthiaceae	1	1			1	1	100
Malpighiales	Euphorbiaceae	6	6	1	1	7	7	100
Malpighiales	Hypericaceae	7	7			7	7	100
Malpighiales	Linaceae	1	1			1	1	100
Malpighiales	Salicaceae	14	14			14	14	100
Malpighiales	Violaceae	6	6	2	2	8	8	100
Malvales	Cistaceae	1	1			1	1	100
Malvales	Malvaceae	13	11			13	11	85
Malvales	Thymelaeaceae	2	1			2	1	50
Myrtales	Lythraceae	1	1			1	1	100
Myrtales	Myrtaceae	3	2			3	2	67
Myrtales	Onagraceae	11	10			11	10	91
Nymphaeales	Cabombaceae	1	1			1	1	100
Nymphaeales	Nymphaeaceae	1	1			1	1	100
Oxalidales	Oxalidaceae	2	2			2	2	100
Pinales	Araucariaceae	1	1			1	1	100
Pinales	Cupressaceae	3	3			3	3	100
Pinales	Pinaceae	3	3			3	3	100
Pinales	Taxaceae	1	1			1	1	100
Piperales	Aristolochiaceae	1	0			1	0	0
Poales	Cyperaceae	44	0			44	0	0
Poales	Juncaceae	23	23			23	23	100
Poales	Poaceae	96	88	7	7	96	88	92
Poales	Typhaceae	4	4			4	4	100
Polypodiales	Aspleniaceae	1	0			1	0	0
Polypodiales	Pteridaceae	1	1			1	1	100
Proteales	Platanaceae	1	1			1	1	100
Ranunculales	Berberidaceae	1	1			1	1	100
Ranunculales	Papaveraceae	6	6	2	2	8	8	100
Ranunculales	Ranunculaceae	19	18			19	18	95
Rosales	Cannabaceae	2	2			2	2	100
Rosales	Moraceae	1	1			1	1	100
Rosales	Rhamnaceae	1	1			1	1	100
Rosales	Rosaceae	65	61			65	61	94
Rosales	Ulmaceae	2	2			2	2	100
Rosales	Urticaceae	3	3	1	1	3	3	100
Salviniales	Azollaceae	1	0			1	0	0
Santalales	Thesiaceae	1	1			1	1	100
Santalales	Viscaceae	1	0			1	0	0
Sapindales	Aceraceae	1	1			1	1	100
Sapindales	Anacardiaceae	1	1			1	1	100
Sapindales	Simaroubaceae	1	1			1	1	100
Saxifragales	Crassulaceae	6	4			6	4	67
Saxifragales	Haloragaceae	1	1			1	1	100
Saxifragales	Saxifragaceae	13	13			13	13	100
Selaginellales	Selaginellaceae	1	1			1	1	100
Solanales	Convolvulaceae	5	5	1	1	5	5	100
Solanales	Solanaceae	8	8			8	8	100
Vitales	Vitaceae	1	0			1	0	0
Total species		972	868	43	43	986	880	89
Genera		560	520	38	38	561	523	93
Families		121	113	17	17	121	113	93

Primer matches are where primers fit with a maximum of 3 bp mismatches and no mismatches in the last two bp at the 3 prime end. Data presented here are from sequences where both primer binding sites were available for analysis; details of species tested for forward and reverse primer matches separately are given in Supplementary Table S1b,c.

Once we had removed duplicate sequences from the same species within our combined database, taxonomic resolution of the ITS2 region was 86.1%, 99.4% and 99.9% at the species, genus and family levels, respectively (n = 1578 species, 821 genera, 154 families). Two species could not be differentiated at the family level: both were ferns. All Mauritian species could be differentiated at the genus and family levels and just two *(Fimbristylis littoralis and F. cymosa)* could not be differentiated at the species level. From UK species, two (1.2%), ten (1.2%) and 221 (14%) species could not be differentiated at the family, genus and species levels respectively.

### *In vitro* testing of primers

We established that the UniPlantF (5′-TGTGAATTGCARRATYCMG-3′) and UniplantR (5′-CCCGHYTGAYYTGRGGTCDC-3′) primers had the greatest amplification success on a subset of plant species (Supplementary Table S2), so only these primers were selected for further *in vitro* and *in silico* testing. *In vitro*, this primer pair successfully amplified 99% of the 169 Mauritian species (Table 2), and 100% of 33 UK species tested (Supplementary Table S3b).

Mock community testing showed that plant species with both long and short amplicon lengths were always coamplified in the same PCR mix, even when there was a bias towards short fragment lengths in the PCR (Supplementary Table S4). Generalised linear mixed effects models indicated that there was a significant association between PCR product concentration and the interaction between treatment (ratio of long and short amplicons) and amplicon length (conditional R-squared = 0.42, f = 9.7504, P = < 0.001). Specifically, when there was a bias in the PCR mix towards long amplicons, the DNA concentration of long amplicons was higher than that of short. The opposite was true when there was a bias towards short amplicons. When there were equal short and long amplicons, the DNA concentration of short amplicons was slightly higher, but this was not significant (Supplementary Fig. S1).

### Threshold analysis

**Figure 2:**
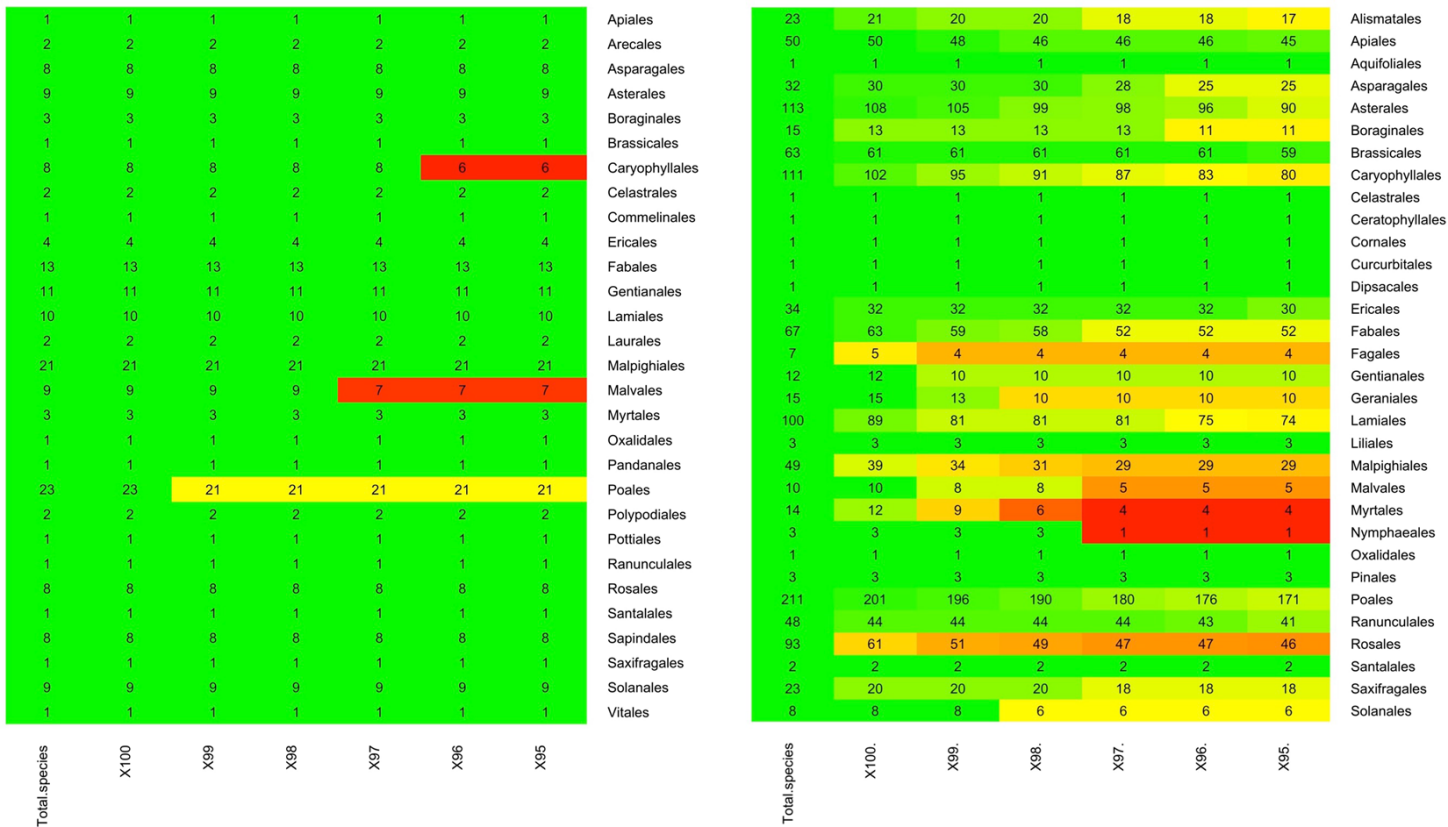
Order-level summary of clustering thresholds for the ITS2 region only between 95 and 100% for (**a**) Mauritius, n = 165 species and (**b**) UK databases, n = 1116 species. Order names are listed on the y-axis and clustering threshold forms the x-axis. The colour of the cells represents the percentage of species within an order that can be identified to species level at a given clustering threshold; numbers within cells show the number of species that can be resolved at each threshold. Colour gradient from green through to red signifies high species-level resolution moving towards poor species-level resolution.

At a 100% clustering threshold, the majority of species tested (n = 1116 in the UK and n = 165 in Mauritius where multiple haplotypes were present in our databases; Fig. 2) could be identified to the species level, although multiple haplotypes were present for many species. As the threshold dropped, the number of species for which taxonomic resolution was possible started to decrease; however, multiple haplotypes for some species remained (Fig. 2). The effect of reducing the clustering threshold differed between families, particularly reducing power of taxonomic resolution in Caryophyllaceae, Myrtales, Poales and Rosales, even at high clustering thresholds (Fig. 2, Supplementary Fig. S2).

### Dietary Case Study 1: Stock Doves

We present sequence read numbers at distinct stages of the bioinformatics pipeline as supplementary information (Supplementary Note S2), as these data are also presented elsewhere^40^ and only a subset is presented here. 5.4% of our sequences matched fungi and bacteria (64 of 1192 unique sequences remaining prior to BLAST matching). We recovered 25 plant species from 13 stock dove samples, with an additional 11 taxa identified to genus level and 4 taxa identified to family level (overall from 31 genera and 18 families; mean ± SE 7.62 ± 0.94 taxonomic units per sample; Supplementary Table S5a; Data S2)^40^. No vertebrate DNA was recovered. When examining the potential for preferential amplification of shorter fragments by comparing amplicon lengths from our NGS run to those from our reference database, we found plant amplicons from the NGS run to be significantly shorter than those within the UK reference database (Mann-Whitney-Wilcoxon, w = 352710, p < 0.001; Fig. 1a).

### Dietary Case Study 2: Telfair’s Skinks

For this dataset, a comprehensive DNA barcode library was available for assigning Illumina reads to taxa^39^. Overall, we recovered and identified 76 plant taxa from Telfair’s skink faecal samples (after removing taxa that do not grow on the study island and were present, for example, because they were kitchen waste composted by the field staff; mean ± SE 5.77 ± 0.16 taxa per sample; Supplementary Table S5b; Data S3). These included species in families for which *in silico* analysis suggested poor primer match (full list of species amplified is provided in Supplementary Table S5). No Telfair’s skink DNA was amplified and sequenced. From the plant species consumed that were also present in the DNA barcode library, 100% could be identified to species (Supplementary Data S3). Of those six consumed species that were absent in the library (Supplementary Data S3), 67% were identified to genus and 33% to species. Overall, this equates to 95% and 5% taxonomic resolution at the species and genus levels respectively. Combining results from the two MiSeq runs within which Telfair’s skink samples were present, 4% of unique sequences were identified as fungi. When examining the potential for preferential amplification of shorter fragments by comparing amplicon lengths from our NGS run to those from our reference database, plant amplicons from both NGS runs were significantly shorter than those within the Mauritius reference database (Mann-Whitney-Wilcoxon, Pool 1: w = 126390, p < 0.001, Pool 2: w = 99468, p < 0.001; Fig. 1b,c).

## Discussion

Current approaches to molecular analysis of herbivory are generally unable to identify the majority of plants to the species level across a range of families, using amplicons short enough to detect degraded DNA recovered from faecal samples. The most widely applied DNA barcode currently used to study herbivory, the P6 loop of the chloroplast *trn*L (UAA) gene, has nearly universal priming sites allowing extremely high taxonomic coverage^22^, and allows about 50% of taxa to be identified to species^27^. However, taxonomic resolution can vary, depending on the local plant community and quality of the reference DNA barcode library: other studies using this region report species level taxonomic assignment of 29.8%^33^ to 77%^34^. Using *trn*L does have the advantage of being able to work with particularly degraded DNA where short amplicons might be expected to be more reliably amplified (12–134 bp using primer pair g and h^18^). By contrast, our new ITS2 primers produce amplicons of 187–387 bp in length, with taxonomic coverage of at least 88%, and taxonomic resolution at the species level as high as 86.1% from *in silico* analyses of three databases. In practice, when used in conjunction with a comprehensive DNA barcode library, taxonomic resolution at the species level can be as high as 100% as shown in our Telfair’s skink case study. Our two case studies demonstrate that these primers successfully amplify DNA from degraded faecal samples from birds and reptiles, and co-amplify multiple plant species from a range of genera and families. Studying trophic interactions between plants and animals at such a fine taxonomic resolution is likely to deepen our knowledge of species ecology and ecosystem dynamics. For example, we have used these primers to provide new insights into the feeding ecology of a declining species, the European turtle dove, including dietary competition with other columbids^40^. We have also used the primers to examine the impacts of ecological replacement^39^. Beyond such dietary studies, the primers also have the potential to inform pollination and seed dispersal networks.

Such high taxonomic resolution is only possible when the sequences for the available plant species are available in a reference DNA barcode library^27^. Indeed, a major criticism of ITS2 has been the lack of reference sequences available for this region^24^. However, the latest update to the ITS2 database has doubled the number of reference sequences available to 711,172, of which 208,822 belong to the Chloroplastida^41^. When sequences are not available for plant species within the study area in question, we strongly suggest that building a study-specific DNA barcode library is invaluable.

There are three further potential criticisms of the use of ITS2 as a DNA barcode^24^. Firstly, there are sometimes paralogous ITS copies present within an individual genome^24,37,42^. From examination of our databases, our threshold analyses and our NGS datasets, this phenomenon appears to be widespread across multiple plant orders; however, this did not hinder taxonomic assignment using a closest match approach. Secondly, amplifying ITS can be difficult with universal primers^37^; however, we found this problem to largely be overcome by amplifying ITS2 only^35,37^, and our primers give good taxonomic coverage. The final criticism is the risk of fungal contamination, given the similarity between plant and fungi universal primer sites within this region^36^. However, we found fungi and bacteria formed only 5.4% of sequences within our UK NGS run, and 4% across our two Mauritian NGS runs. These figures are slightly higher than that of 2–3% suggested previously from *in silico* searches^37^, but after discarding fungal sequences we retained more than sufficient plant read depth for our herbivory analyses. As our primers produce a range of amplicon sizes that differ between plant families, we examined the potential for size bias in our NGS datasets compared to our databases of available species in each region^43^. Overall, UK NGS sequences were significantly shorter than those expected from the reference database, although this is likely to be due to 235 polymorphic sequences of below average (262 bp) length, all assigned to *Brassica* species, which are known to show high within-species diversity at the ITS regions^44^, and were present in all of our stock dove samples. Mauritian sequences from both pools were both significantly shorter than from the reference database; however, sequences of 331 bp (the length of the longest sequence in the reference database) were recovered from both pools. However, these results may be due to dietary preferences of the two consumers rather than size bias. Our mock community testing indicated that long fragments are always amplified, even when there is a bias in the PCR mix towards shorter fragments. Overall, the concentration of PCR products varied as would be expected: when there were more short fragments in the PCR, the concentration of short was higher than that of long amplicons and the reverse was true when there was a bias towards long fragments in the PCR mix. This indicates that size bias, at the PCR stage, may not be a significant for this primer set, especially when read number is not used to quantify diet. Given the findings from our threshold analysis, that intraspecific variation at the ITS2 region will not be removed by clustering into MOTUs without losing taxonomic resolution, we recommend a closest species match approach to sequence identification^45,46^, rather than a MOTU clustering approach, if the aim of the study is to identify specific dietary components. This also removes any issues caused by potential multiple ITS polymorphisms within an individual^47^ but does emphasise the need for comprehensive reference barcode libraries for the study system. If such a reference barcode library is not available then a clustering approach to examine, for example, dietary niche partitioning, may be more appropriate. Sanger sequencing of multiple samples from individual plant species may not adequately represent total ITS diversity due to low-frequency polymorphisms^47^ (in, for example, Brassicaceae^44^), as this may only result in the most frequent polymorphism being detected. In such cases it may be useful to include some single species plant samples in an NGS run alongside faecal DNA for analysis, to assist reliable species assignment of multiple polymorphisms.

Our *in vitro* and *in silico* testing of the UniPlant primers proved that they can amplify a diverse assemblage of plants. The *in silico* PCR results were more conservative than the *in vitro* testing. For example, *in silico* testing revealed that the primers were a poor fit for species within the Orchidaceae and Cyperaceae families, but these were shown to amplify successfully *in vitro*. Indeed, our detailed Telfair’s skink data show *Cyperus dubius* (Cyperaceae) to be co-amplified in 16% of faecal samples, alongside a range of other plant species with better primer fit. Thus, in practice, the primers are clearly better than suggested by the *in silico* results. However, such species with potentially poor primer fit should be tested *in vitro* to confirm successful amplification before use for the examination of herbivory. Future studies using our primers may also benefit from including known mixtures of DNA samples to ensure co-amplification of likely plant DNA combinations from the relevant study system. In practice different plant species eaten by a generalist herbivore will inevitably be amplified to different degrees, regardless of the primers selected, which is why we base our analyses on frequency of occurrence within faecal extracts, rather than numbers of sequences generated by NGS. Different plant species will also be digested to different degrees, and the number of copies of the target gene per cell will vary with species, making frequency of occurrence the most reliable quantitative measure.

Our novel primers amplify a fragment of 187–387 bp, which is suitable for use with NGS platforms, and here we show that they are general enough to amplify the vast majority of the phylogenetically diverse array of plant species found in the UK and Mauritius, and therefore highly likely to be equally useful in other parts of the globe. We recommend *in silico* followed by *in vitro* testing of likely dietary items, particularly if they are ferns or within the Cyperaceae, Orchidaceae, Hydrocharitaceae or Thymelaeaceae families. A comprehensive DNA barcode reference library is invaluable to obtain high taxonomic resolution, and to avoid the potential pitfall of setting a clustering threshold, permitting accurate assignment of taxa based on a closest match approach.

## Methods

### Barcode databases

#### Mauritian database

Plant tissue samples were collected from two Mauritian islands (Ile aux Aigrettes and Round Island) as part of a larger study in which we DNA barcoded the plant communities in order to examine herbivory by introduced and native reptiles and birds^39^. Plant identity was verified prior to DNA barcoding to ensure taxonomic accuracy. Eighty-four sequences available at an early stage of the work were used for primer design (Supplementary Table S3a). *In vitro* primer testing was carried out on DNA samples from 169 species from 65 families. *In silico* analyses were carried out on a dataset of 464 sequences, 167 species and 63 families (of which eight were downloaded from GenBank to supplement field collected samples and form a complete barcode library).

#### UK database

6054 ITS2 sequences from 1651 UK plant species from 151 families were downloaded from GenBank. These largely, but not entirely, consisted of vouchered sequences from a comprehensive analysis of the ITS2 region of UK plants (de Vere *et al*., unpubl. data). Where possible, if sequences did not span both priming sites we obtained untrimmed sequences. Where available from GenBank, this included at least one representative from each genus of plants listed on the Ecological Database of the British Isles^48^ (a comprehensive list of both native and introduced plant species found in the UK). We downloaded a maximum of one sequence per species from GenBank, so where multiple haplotypes of a species are present within the database the majority of these are from vouchered specimens. Synonyms were checked with The Plant List^49^.

#### UK columbid database

Thirty six UK plant species were collected and barcoded as part of a separate study examining the diet of UK columbids, with a focus on European turtle doves^40^, with an additional 14 species represented in the database by sequences downloaded from GenBank. This included 31 species previously identified in the diet of turtle doves using microscopy, seven species known to be present within commercial seed mixes and 12 additional species commonly found on arable farmland (Supplementary Table S3b). Thirty three of these sequences (those available at an early stage of this work) were used for primer design and *in vitro* testing.

### Generation of Reference Databases

DNA extractions were carried out either following Randall *et al*.^50^ after samples were ground under liquid nitrogen, or using the Qiagen DNeasy plant kit (Qiagen, Manchester, UK). The complete second internal transcribed spacer of nuclear ribosomal DNA (ITS2) and partial 5.8S and 26S sequences were amplified using primer pair S2F and S3R^35^. Where amplification with this primer pair failed, a second ITS2 primer pair were tried, ITS-p3 and ITS-p4^38^. PCRs were carried out in 10 µL reaction volumes containing 2 µL DNA template, 1x PCR buffer, 2.0 mM MgCl_2_, 0.2 µM of each primer (at 10 mM), 0.2 mM of each dNTP and 1 U Go Taq Flexi (Promega, Southampton, UK). For problematic samples, a multiplex PCR mix (Qiagen, Manchester, UK) was used, with primers and DNA at the same concentration and volume described above. Reaction conditions were an initial denaturation step at 95 °C for 10 min, followed by 40 cycles of 95 °C for 30 s, 56 °C for 30 s and 72 °C for 1 min, and a final extension of 72 °C for 10 min. PCR products were sequenced in both directions by Eurofins Genomics (Wolverhampton, UK). Contigs were constructed and consensus sequences created in Sequencher version 5.4.6^51^ or MEGA6^52^ after manually editing sequences. Consensus sequences were aligned using automated ClustalW alignment in BioEdit^53^ or ClustalX^54^, for *in silico* analysis (see below).

### Short amplicon primer design for diet analysis and *in vitro* testing

**Figure 3:**
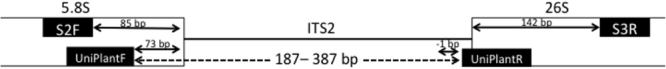
Schematic diagram of priming sites within the second internal transcribed spacer (ITS2) and flanking regions (5.8S and 26S). The location of S2F and S3R priming sites^35^ are shown alongside UniPlantF and UniplantR from this study. The distances of the priming sites from the ITS2 region are shown (bp). Distances are based on a representative *Asparagus setaceus* sequence (NCBI Accession number KY700230). S2F and UniPlantF overlap by 7 bp. UniPlantR begins on the last 1 bp of ITS2 and continues into 26S. The amplicon size range, across all sequences assessed in this study, of the UniPlant primers is shown. Schematic not to scale.

A subset of aligned ITS2 and partial 26S and 5.8S sequences (Supplementary Table S3a,b; UK columbid database n = 33, Mauritius database n = 84) were used to design primers for a short ITS2 amplicon to maximise amplification from the degraded DNA found in faecal samples (Fig. 3). Aligned sequences were examined by eye in MEGA6^52^ in order to detect suitably conserved sites. Five forward and seven reverse primers were designed and tested *in vitro* on a subset of plant DNA from key dietary items (mean ± SE: 14.8 ± 10.2 plant DNA samples per primer pair; Supplementary Table S2). All *in vitro* testing involved amplification in 10 µL PCR reaction volumes with reagents and template DNA in the same concentrations as described above. Reaction conditions were also the same as above, after initially testing annealing temperatures from 46 °C–56 °C by gradient PCR. Successful amplification was determined by visualisation on a 2% agarose gel stained with SYBR^®^Safe (ThermoFisher Scientific, Paisley, UK). Primers that failed initial tests (amplification failure, faint bands, multiple banding) on a small number of plant DNA samples were rejected with no further testing (Supplementary Table S2). These initial *in vitro* tests revealed that one primer pair, UniPlantF and UniPlantR, had the highest amplification success so these were subjected to further *in vitro* testing against all available Mauritian plant species and the field-collected UK species.

To determine whether the primers preferentially amplified those plant species with shorter ITS2 fragments over those with longer fragments, we assembled 15 mock communities from plant tissue DNA extracts. Each mock community contained six plant species each at an initial concentration of 0.3 ng/µL before adding to the PCR mix but the ratio of those plant species with long or short amplicons varied across three treatments: an equal treatment of 3 long and 3 short plant species, a bias towards short fragments containing 2 long and 4 short species, a bias towards long fragments containing 4 long and 2 short species. Plant species with ITS2 amplicon lengths using the UniPlant primers of between 267 and 280 bp were classified as short, and between 310 and 336 were classified as long. PCRs were carried out in 10 µL reaction volumes with a total DNA concentration of 0.3 ng/µL with reagent concentrations and PCR reaction conditions identical to those used in Case Study 2 (see below). PCR products were analysed by high-resolution capillary electrophoresis using a QIAxcel (Qiagen, Manchester, UK) to determine the DNA concentration of the long and short amplicons. Whether DNA concentration was significantly associated with amplicon length, treatment or their interaction was analysed using generalised linear mixed effects models in the lme4^55^ package in R^56^. Amplicon length and treatment were modelled as fixed effects and PCR reaction was included as a random effect with DNA concentration as the dependent variable. The model was run using the Gaussian error structure and the identity link function on normal data. Model assumptions were checked by examining the standardised residuals.

### *In silico* testing

To further test the suitability of this primer pair, *in silico* PCR was carried out on a larger number of species from all three databases using ecoPCR within OBITools^57^. We allowed for a maximum of three base mismatches per primer ensuring the last two bases at the 3′ end were an exact match^58^, specifying a minimum amplicon length of 100 bp and a maximum of 500 bp. Where DNA sequences did not encompass both forward and reverse priming sites, primers were tested independently and reported in the supplementary information (Supplementary Table S1a,b,c). To examine the potential for preferential amplification of short-length amplicons^43^, we calculated mean amplicon length per family from the ecoPCR output and compared the amplicon distribution of each of the UK and Mauritius databases to the NGS data from our UK and Mauritian studies (see below). We used Mann-Whitney-Wilcoxon tests to allow for non-normal distribution of amplicon lengths.

We define taxonomic resolution as per Pompanon *et al*.^18^, as the percentage of taxa unambiguously identified for a given taxonomic level. To test the taxonomic resolution of the ITS2 region within the UniPlant amplicon (Fig. 1), we combined all three databases and removed identical sequences derived from the same species and those sequences of poor quality (resulting in 3550 total sequences, representing 1659 species, 828 genera and 155 families). We used the ITSx software^59^ to extract the ITS2 region from our amplicons to form our ITS2 database (ITS2 successfully extracted from 2216 sequences, representing 1577 species, 821 genera and 143 families). We used the “derep_prefix” command in USEARCH^60^ to identify identical sequences within each database; we then calculated the number of taxa within which multiple species had identical ITS2 sequences.

### Testing clustering thresholds

To test whether sequences resulting from NGS analysis of faecal samples using our primers should be clustered into MOTUs within the bioinformatics pipeline, and if so at what threshold, we used reference sequences from both the Mauritian (n = 167 species and 464 sequences) and UK databases (n = 1116 species and 2619 sequences) from species where multiple vouchered sequences were available. We ran the sequence files through the USEARCH^60^ command “cluster_fast” with an identity threshold of 95%. We then used the percentage similarity values between clustered sequences from the cluster format output file to identify, for cut-offs between 95 and 100%, how many different species and haplotypes would be clustered together. Resolution at each clustering threshold is displayed as heat maps, at the order level. Heat maps were created using the “heatmap.2” function in the *gplots* package^61^ in R^56^.

### Dietary case studies

These primers were originally designed for dietary analysis in two separate studies: one assessing the diet of Pink Pigeons *Nesoenas mayeri*, Telfair’s skinks and Aldabra giant tortoises *Aldabrachelys gigantea* in Mauritius; and one investigating the diet of UK doves and pigeons (turtle dove, collared dove *Streptopelia decaocto*, woodpigeon *Columba palumbus* and stock dove). Detailed results for these two studies will be published elsewhere^39,40^), but to demonstrate the effectiveness of our primers on faecal samples, we present comprehensive data from one species from each study (stock dove: Case Study 1; Telfair’s skinks: Case Study 2) here. Detailed methods for sample collection, laboratory protocols and data analyses are provided in Supplementary Note S1.

### Data availability

New accession numbers for sequences generated from this study, and those used in our databases are provided in the Supplementary Information, along with our detailed case study data. Raw MiSeq data from the UK columbid case study is available on the NCBI Sequence Read Archive under accession number SRP136381, and detailed individual level taxonomic unit presence-absence data are available from JCD upon reasonable request. Raw MiSeq data from the Mauritian study will be deposited in the NCBI Sequence Read Archive upon acceptance.

### Accession numbers

DNA sequences: available on GenBank under the accession numbers listed in Supplementary Data S1. Raw MiSeq data from the UK columbid case study is available on the NCBI Sequence Read Archive under accession number SRP136381, and detailed individual level taxonomic unit presence-absence data are available from JCD upon reasonable request. Raw MiSeq data from the Mauritian study will be deposited in the NCBI Sequence Read Archive upon acceptance.

## Electronic supplementary material

Supplementary Information

Supplementary Data 2

Supplementary Data 3
