# Supplementary material for: New universal ITS2 primers for high-resolution herbivory analyses using DNA metabarcoding in both tropical and temperate zones
Source: Sci Rep. 2018 Jun 4;8:8542. doi: 10.1038/s41598-018-26648-2 (PMC5986805; doi:10.1038/s41598-018-26648-2)
Supplement: Supplementary file 1 — Supplementary Information [file 41598_2018_26648_MOESM1_ESM.pdf]

Supplementary Information

New universal ITS2 primers for high-resolution herbivory analyses using DNA metabarcoding in both tropical and temperate zones

Rosemary J. Moorhouse-Gann, Jenny C. Dunn, Natasha de Vere, Martine Goder, Nik Cole, Helen Hipperson, William O. C. Symondson

Supplementary Table S1a. Results of *in silico* analysis of primer fit for UniPlantR for Mauritian plant families where forward primer fit could not be tested due to short sequence lengths. Matches are where primers fit with a maximum of 3bp mismatches and no mismatches in the last two bp at the 3 prime end. For all Mauritian sequences, if the UniPlantF priming site was present, the UniplantR priming site was also present (Table 2).

Order	Family	No. species where reverse primer only tested *in silico*	No. species where reverse primer matches *in silico*	% matches reverse primer only
Apiales	Araliaceae	1	1	100
Arecales	Arecaceae	2	2	100
Asparagales	Amaryllidaceae	1	1	100
Asparagales	Orchidaceae	2	0	0
Asparagales	Xanthorrhoeaceae	1	1	100
Asterales	Asteraceae	6	6	100
Asterales	Goodeniaceae	1	1	100
Boraginales	Boraginaceae	3	3	100
Brassicales	Caricaceae	1	1	100
Caryophyllales	Aizoaceae	1	1	100
Caryophyllales	Amaranthaceae	1	1	100
Caryophyllales	Nyctaginaceae	1	1	100
Caryophyllales	Petiveriaceae	1	1	100
Caryophyllales	Portulacaceae	1	1	100
Celastrales	Celastraceae	2	2	100
Commelinales	Commelinaceae	1	1	100
Ericales	Ebenaceae	2	2	100
Ericales	Lecythidaceae	1	1	100

Order	Family	No. species where reverse primer only tested *in silico*	No. species where reverse primer matches *in silico*	% matches reverse primer only
Ericales	Sapotaceae	1	1	100
Fabales	Fabaceae	10	7	70
Gentianales	Apocynaceae	6	4	67
Gentianales	Rubiaceae	4	4	100
Lamiales	Acanthaceae	1	1	100
Lamiales	Bignoniaceae	1	1	100
Lamiales	Lamiaceae	1	1	100
Lamiales	Lauraceae	1	1	100
Lamiales	Oleaceae	2	1	50
Lamiales	Scrophulariaceae	1	1	100
Lamiales	Verbenaceae	2	2	100
Malpighiales	Erythroxylaceae	1	1	100
Malpighiales	Euphorbiaceae	6	6	100
Malpighiales	Passifloraceae	2	1	50
Malpighiales	Phyllanthaceae	8	5	63
Malpighiales	Salicaceae	3	3	100
Malvales	Malvaceae	8	5	63
Malvales	Thymelaeaceae	1	1	100
Myrtales	Combretaceae	1	1	100
Myrtales	Lythraceae	1	1	100
Myrtales	Myrtaceae	1	1	100
Poales	Cyperaceae	3	0	0
Poales	Poaceae	17	12	71
Polypodiales	Pteridaceae	1	0	0

Order	Family	No. species where reverse primer only tested *in silico*	No. species where reverse primer matches *in silico*	% matches reverse primer only
Ranunculales	Papaveraceae	1	1	100
Rosales	Moraceae	3	2	67
Rosales	Rhamnaceae	3	2	67
Rosales	Urticaceae	1	1	100
Santalales	Santalaceae	1	1	100
Sapindales	Anacardiaceae	1	1	100
Sapindales	Burseraceae	1	1	100
Sapindales	Rutaceae	1	0	0
Sapindales	Sapindaceae	2	1	50
Saxifragales	Crassulaceae	1	1	100
Solanales	Convolvulaceae	2	2	100
Solanales	Solanaceae	3	3	100
Vitales	Vitaceae	1	1	100
**Totals**		**132**	**104**	**79**

Supplementary Table S1b. Results of *in silico* analysis of primer fit for UniPlantF for plant families across both UK databases, where UniPlantR primer fit could not be tested due to short sequence lengths.

		UK database		UK Columbid database		Overall		
Order	Family	F tested	F matches	F tested	F matches	F tested	F matches	% match
Lamiales	Acanthaceae	1	1			1	1	100
Sapindales	Aceraceae	1	1			1	1	100
Acorales	Acoraceae	1	1			1	1	100
Dipsacales	Adoxaceae	4	4			4	4	100
Caryophyllales	Aizoaceae	3	3			3	3	100
Alismatales	Alismataceae	7	5			7	5	71
Liliales	Alstroemeriaceae	1	1			1	1	100
Caryophyllales	Amaranthaceae	1	1			1	1	100
Asparagales	Amaryllidaceae	10	9			10	9	90
Sapindales	Anacardiaceae	1	1			1	1	100
Apiales	Apiaceae	52	49	1	1	52	49	94
Alismatales	Aponogetonaceae	1	1			1	1	100
Aquifoliales	Aquifoliaceae	1	1			1	1	100
Alismatales	Araceae	4	4			4	4	100
Apiales	Araliaceae	4	4			4	4	100
Pinales	Araucariaceae	1	1			1	1	100
Piperales	Aristolochiaceae	2	1			2	1	50
Asparagales	Asparagaceae	5	4			5	4	80
Polypodiales	Aspleniaceae	1	0			1	0	0
Asterales	Asteraceae	122	115	6	6	122	115	94
Salviniales	Azollaceae	1	1			1	1	100
Ericales	Balsaminaceae	1	1			1	1	100
Ranunculales	Berberidaceae	1	1			1	1	100
Fagales	Betulaceae	6	6			6	6	100

		UK database		UK Columbid database		Overall		
Order	Family	F tested	F matches	F tested	F matches	F tested	F matches	% match
Boraginales	Boraginaceae	22	22			22	22	100
Brassicales	Brassicaceae	75	70	3	3	76	71	93
Alismatales	Butomaceae	1	1			1	1	100
Buxales	Buxaceae	2	2			2	2	100
Nymphaeales	Cabombaceae	1	1			1	1	100
Lamiales	Calceolariaceae	1	1			1	1	100
Asterales	Campanulaceae	13	13			13	13	100
Rosales	Cannabaceae	2	2			2	2	100
Dipsacales	Caprifoliaceae	12	12			12	12	100
Caryophyllales	Caryophyllaceae	53	51	6	6	54	52	96
Celastrales	Celastraceae	3	3			3	3	100
Ceratophyllales	Ceratophyllaceae	2	2			2	2	100
Caryophyllales	Chenopodiaceae	24	21	2	2	24	21	88
Malvales	Cistaceae	2	2			2	2	100
Liliales	Colchicaceae	1	1			1	1	100
Solanales	Convolvulaceae	5	5	1	1	5	5	100
Cornales	Cornaceae	1	1			1	1	100
Saxifragales	Crassulaceae	6	6			6	5	83
Cucurbitales	Cucurbitaceae	5	5			5	5	100
Pinales	Cupressaceae	8	8			8	8	100
Alismatales	Cymodoceaceae	1	1			1	1	100
Poales	Cyperaceae	50	44			50	44	88
Polypodiales	Dennstaedtiaceae	1	1			1	1	100
Ericales	Diapensiaceae	1	1			1	1	100
Caryophyllales	Droseraceae	2	2			2	2	100
Rosales	Elaeagnaceae	2	2			2	2	100
Equisetales	Equisetaceae	1	1			1	1	100

		UK database		UK Columbid database		Overall		
Order	Family	F tested	F matches	F tested	F matches	F tested	F matches	% match
Ericales	Ericaceae	21	20			21	20	95
Malpighiales	Euphorbiaceae	6	6	1	1	7	7	100
Fabales	Fabaceae	68	64	5	5	71	67	94
Fagales	Fagaceae	3	3			3	3	100
Garryales	Garryaceae	1	1			1	1	100
Gentianales	Gentianaceae	8	8			8	8	100
Geraniales	Geraniaceae	15	15	1	1	15	15	100
Lamiales	Gesneriaceae	1	1			1	1	100
Apiales	Griseliniaceae	1	1			1	1	100
Saxifragales	Grossulariaceae	1	1			1	1	100
Gunnerales	Gunneraceae	1	1			1	1	100
Saxifragales	Haloragaceae	1	1			1	1	100
Sapindales	Hippocastanaceae	1	1			1	1	100
Asparagales	Hyacinthaceae	3	3			3	3	100
Cornales	Hydrangeaceae	3	2			3	2	67
Alismatales	Hydrocharitaceae	8	8			8	8	100
Boraginales	Hydrophyllaceae	1	1			1	1	100
Malpighiales	Hypericaceae	7	7			7	7	100
Asparagales	Iridaceae	3	3			3	3	100
Isoetales	Isoetaceae	1	1			1	1	100
Fagales	Juglandaceae	1	1			1	1	100
Poales	Juncaceae	23	17			23	17	74
Alismatales	Juncaginaceae	1	1			1	1	100
Lamiales	Lamiaceae	28	27			28	27	96
Laurales	Lauraceae	1	1			1	1	100
Lamiales	Lentibulariaceae	4	3			4	3	75
Liliales	Liliaceae	7	6			7	6	86

		UK database		UK Columbid database		Overall		
Order	Family	F tested	F matches	F tested	F matches	F tested	F matches	% match
Brassicales	Limnanthaceae	1	1			1	1	100
Malpighiales	Linaceae	3	3			3	3	100
Lycopodiales	Lycopodiaceae	1	1			1	1	100
Myrtales	Lythraceae	1	1			1	1	100
Malvales	Malvaceae	15	15			15	15	100
Liliales	Melanthiaceae	1	1			1	1	100
Asterales	Menyanthaceae	2	2			2	2	100
Caryophyllales	Montiaceae	2	2			2	2	100
Rosales	Moraceae	2	2			2	2	100
Fagales	Myricaceae	1	1			1	1	100
Myrtales	Myrtaceae	3	3			3	3	100
Fagales	Nothofagaceae	1	1			1	1	100
Nymphaeales	Nymphaeaceae	2	2			2	2	100
Lamiales	Oleaceae	5	5			5	5	100
Myrtales	Onagraceae	15	14			15	14	93
Asparagales	Orchidaceae	26	23			26	23	88
Lamiales	Orobanchaceae	25	25			25	25	100
Osmundales	Osmundaceae	1	1			1	1	100
Oxalidales	Oxalidaceae	2	1			2	1	50
Saxifragales	Paeoniaceae	1	1			1	1	100
Ranunculales	Papaveraceae	13	13	2	2	15	15	100
Lamiales	Paulowniaceae	1	1			1	1	100
Lamiales	Phrymaceae	1	1			1	1	100
Caryophyllales	Phytolaccaceae	1	1			1	1	100
Pinales	Pinaceae	8	8			8	8	100
Apiales	Pittosporaceae	1	1			1	1	100
Lamiales	Plantaginaceae	29	25	2	2	31	27	87

		UK database		UK Columbid database		Overall		
Order	Family	F tested	F matches	F tested	F matches	F tested	F matches	% match
Proteales	Platanaceae	1	1			1	1	100
Caryophyllales	Plumbaginaceae	3	3			3	3	100
Poales	Poaceae	127	121	8	8	127	121	95
Ericales	Polemoniaceae	2	2			2	2	100
Fabales	Polygalaceae	3	3			3	3	100
Caryophyllales	Polygonaceae	14	12	3	3	15	13	87
Caryophyllales	Portulacaceae	1	1			1	1	100
Alismatales	Potamogetonaceae	6	6			6	6	100
Ericales	Primulaceae	10	10	1	1	10	10	100
Polypodiales	Pteridaceae	1	1			1	1	100
Ranunculales	Ranunculaceae	27	27	1	1	27	27	100
Brassicales	Resedaceae	1	1			1	1	100
Rosales	Rhamnaceae	2	2			2	2	100
Rosales	Rosaceae	86	82			86	82	95
Gentianales	Rubiaceae	5	5	1	1	5	5	100
Malpighiales	Salicaceae	14	14			14	14	100
Sapindales	Sapindaceae	1	1			1	1	100
Ericales	Sarraceniaceae	1	1			1	1	100
Saxifragales	Saxifragaceae	18	18			18	18	100
Alismatales	Scheuchzeriaceae	1	0			1	0	0
Lamiales	Scrophulariaceae	6	6			6	6	100
Selaginellales	Selaginellaceae	1	1			1	1	100
Sapindales	Simaroubaceae	1	1			1	1	100
Solanales	Solanaceae	12	12			12	12	100
Caryophyllales	Tamaricaceae	1	1			1	1	100
Pinales	Taxaceae	1	1			1	1	100
Santalales	Thesiaceae	1	1			1	1	100

		UK database		UK Columbid database		Overall		
Order	Family	F tested	F matches	F tested	F matches	F tested	F matches	% match
Malvales	Thymelaeaceae	2	1			2	1	50
Alismatales	Tofieldiaceae	1	1			1	1	100
Brassicales	Tropaeolaceae	1	1			1	1	100
Poales	Typhaceae	4	4			4	4	100
Rosales	Ulmaceae	3	3			3	3	100
Rosales	Urticaceae	3	3	1	1	3	3	100
Lamiales	Verbenaceae	1	1			1	1	100
Malpighiales	Violaceae	7	6	2	2	9	8	89
Santalales	Viscaceae	1	0			1	0	0
Vitales	Vitaceae	1	1			1	1	100
Asparagales	Xanthorrhoeaceae	2	1			2	1	50
Alismatales	Zosteraceae	1	1			1	1	100
	Total species	1286	1213	47	47	1299	1225	94
	Total genera	824	806	42	42	824	806	98
	Total families	144	141	18	18	144	141	97

Supplementary Table S1c. Results of *in silico* analysis of primer fit for UniPlantR for plant families across both UK databases, where UniPlantF primer fit could not be tested due to short sequence lengths.

		UK Genus level		UK columbid database		Overall		
Order	Family	R tested	R matches	R tested	R matches	R tested	R matches	% match
Lamiales	Acanthaceae	1	1			1	1	100
Sapindales	Aceraceae	1	1			1	1	100
Acorales	Acoraceae	1	1			1	1	100
Dipsacales	Adoxaceae	4	4			4	4	100
Caryophyllales	Aizoaceae	1	1			1	1	100
Alismatales	Alismataceae	6	6			6	6	100
Caryophyllales	Amaranthaceae	5	5			5	5	100
Asparagales	Amaryllidaceae	10	9			10	9	90
Sapindales	Anacardiaceae	1	1			1	1	100
Apiales	Apiaceae	58	55	1	1	58	55	95
Alismatales	Aponogetonaceae	1	1			1	1	100
Aquifoliales	Aquifoliaceae	1	1			1	1	100
Alismatales	Araceae	4	4			4	4	100
Apiales	Araliaceae	3	3			3	3	100
Pinales	Araucariaceae	1	1			1	1	100
Piperales	Aristolochiaceae	1	0			1	0	0
Asparagales	Asparagaceae	4	3			4	3	75
Polypodiales	Aspleniaceae	1	0			1	0	0
Asterales	Asteraceae	122	121	7	7	123	122	99
Salviniales	Azollaceae	1	0			1	0	0
Ericales	Balsaminaceae	2	2			2	2	100
Ranunculales	Berberidaceae	1	1			1	1	100
Fagales	Betulaceae	7	7			7	7	100
Boraginales	Boraginaceae	23	23			23	23	100

		UK Genus level		UK columbid database		Overall		
Order	Family	R tested	R matches	R tested	R matches	R tested	R matches	% match
Brassicales	Brassicaceae	76	73	3	3	77	74	96
Alismatales	Butomaceae	1	1			1	1	100
Buxales	Buxaceae	1	1			1	1	100
Nymphaeales	Cabombaceae	1	1			1	1	100
Lamiales	Calceolariaceae	1	1			1	1	100
Asterales	Campanulaceae	15	14			15	14	93
Rosales	Cannabaceae	2	2			2	2	100
Dipsacales	Caprifoliaceae	7	6			7	6	86
Caryophyllales	Caryophyllaceae	69	68	6	6	69	68	99
Celastrales	Celastraceae	2	2			2	2	100
Ceratophyllales	Ceratophyllaceae	2	2			2	2	100
Caryophyllales	Chenopodiaceae	24	24	1	1	24	24	100
Malvales	Cistaceae	2	2			2	2	100
Solanales	Convolvulaceae	7	7	1	1	7	7	100
Cornales	Cornaceae	1	1			1	1	100
Saxifragales	Crassulaceae	8	6			8	6	75
Cucurbitales	Cucurbitaceae	3	3			3	3	100
Pinales	Cupressaceae	4	4			4	4	100
Alismatales	Cymodoceaceae	1	0			1	0	0
Poales	Cyperaceae	79	0			79	0	0
Ericales	Diapensiaceae	1	1			1	1	100
Caryophyllales	Droseraceae	3	3			3	3	100
Polypodiales	Dryopteridaceae	1	0			1	0	0
Malpighiales	Elatinaceae	1	1			1	1	100
Ericales	Ericaceae	23	22			23	22	96
Malpighiales	Euphorbiaceae	9	9	1	1	10	10	100
Fabales	Fabaceae	76	68	5	5	76	68	89

		UK Genus level		UK columbid database		Overall		
Order	Family	R tested	R matches	R tested	R matches	R tested	R matches	% match
Fagales	Fagaceae	3	3			3	3	100
Gentianales	Gentianaceae	12	12			12	12	100
Geraniales	Geraniaceae	15	15	1	1	15	15	100
Lamiales	Gesneriaceae	1	0			1	0	0
Apiales	Griseliniaceae	1	1			1	1	100
Saxifragales	Grossulariaceae	2	2			2	2	100
Gunnerales	Gunneraceae	1	1			1	1	100
Saxifragales	Haloragaceae	4	4			4	4	100
Asparagales	Hyacinthaceae	2	2			2	2	100
Cornales	Hydrangeaceae	1	1			1	1	100
Alismatales	Hydrocharitaceae	6	3			6	3	50
Boraginales	Hydrophyllaceae	1	1			1	1	100
Malpighiales	Hypericaceae	10	10			10	10	100
Asparagales	Iridaceae	2	2			2	2	100
Fagales	Juglandaceae	1	1			1	1	100
Poales	Juncaceae	31	31			31	31	100
Alismatales	Juncaginaceae	2	2			2	2	100
Lamiales	Lamiaceae	29	27			29	27	93
Lamiales	Lentibulariaceae	5	5			5	5	100
Liliales	Liliaceae	6	6			6	6	100
Malpighiales	Linaceae	3	3			3	3	100
Myrtales	Lythraceae	2	2			2	2	100
Malvales	Malvaceae	13	11			13	11	85
Liliales	Melanthiaceae	1	1			1	1	100
Asterales	Menyanthaceae	2	2			2	2	100
Caryophyllales	Montiaceae	2	2			2	2	100
Rosales	Moraceae	1	1			1	1	100

		UK Genus level		UK columbid database		Overall		
Order	Family	R tested	R matches	R tested	R matches	R tested	R matches	% match
Fagales	Myricaceae	1	1			1	1	100
Myrtales	Myrtaceae	3	2			3	2	67
Nymphaeales	Nymphaeaceae	3	2			3	2	67
Lamiales	Oleaceae	3	3			3	3	100
Myrtales	Onagraceae	15	14			15	14	93
Asparagales	Orchidaceae	28	25			28	25	89
Lamiales	Orobanchaceae	34	34			34	34	100
Oxalidales	Oxalidaceae	2	2			2	2	100
Ranunculales	Papaveraceae	17	17	2	2	17	17	100
Caryophyllales	Phytolaccaceae	1	1			1	1	100
Pinales	Pinaceae	3	3			3	3	100
Apiales	Pittosporaceae	1	1			1	1	100
Lamiales	Plantaginaceae	38	37	2	2	39	38	97
Proteales	Platanaceae	1	1			1	1	100
Caryophyllales	Plumbaginaceae	3	3			3	3	100
Poales	Poaceae	124	122	8	8	125	123	98
Ericales	Polemoniaceae	1	1			1	1	100
Fabales	Polygalaceae	2	2			2	2	100
Caryophyllales	Polygonaceae	17	17	2	2	17	17	100
Polypodiales	Polypodiaceae	1	0			1	0	0
Caryophyllales	Portulacaceae	1	1			1	1	100
Alismatales	Potamogetonaceae	14	14			14	14	100
Ericales	Primulaceae	13	13	1	1	13	13	100
Polypodiales	Pteridaceae	1	1			1	1	100
Ranunculales	Ranunculaceae	34	33			34	33	97
Brassicales	Resedaceae	2	2			2	2	100
Rosales	Rhamnaceae	3	3			3	3	100

		UK Genus level		UK columbid database		Overall		
Order	Family	R tested	R matches	R tested	R matches	R tested	R matches	% match
Rosales	Rosaceae	93	90			93	90	97
Gentianales	Rubiaceae	4	4	1	1	4	4	100
Malpighiales	Salicaceae	16	16			16	16	100
Saxifragales	Saxifragaceae	15	15			15	15	100
Alismatales	Scheuchzeriaceae	1	1			1	1	100
Lamiales	Scrophulariaceae	5	5			5	5	100
Selaginellales	Selaginellaceae	1	1			1	1	100
Sapindales	Simaroubaceae	1	1			1	1	100
Solanales	Solanaceae	10	10			10	10	100
Caryophyllales	Tamaricaceae	1	1			1	1	100
Pinales	Taxaceae	1	1			1	1	100
Santalales	Thesiaceae	1	1			1	1	100
Malvales	Thymelaeaceae	2	2			2	2	100
Alismatales	Tofieldiaceae	1	1			1	1	100
Poales	Typhaceae	4	4			4	4	100
Rosales	Ulmaceae	3	3			3	3	100
Rosales	Urticaceae	3	3	1	1	3	3	100
Lamiales	Verbenaceae	2	2			2	2	100
Malpighiales	Violaceae	12	12	2	2	12	12	100
Santalales	Viscaceae	1	1			1	1	100
Vitales	Vitaceae	1	0			1	0	0
Asparagales	Xanthorrhoeaceae	1	1			1	1	100
Alismatales	Zosteraceae	1	1			1	1	100
	Total species	1385	1255	45	45	1390	1260	91
	Total genera	643	603	40	40	645	605	94
	Total families	128	119	17	17	128	119	93

Supplementary Table S2. Primers designed in this study with initial *in vitro* testing results

Forward primer	Primer sequence 5’-3’	Reverse primer	Primer sequence 5’-3’	No. species tested *in vitro*	Amplification success (%)
TAS1 forward	GCRAGTTGCGCCYVRVK	TAS1 reverse	TGCTTAARCTCRGYGGGTRDY	10	0
		TAS2 reverse	ATATGCTTAARCTCRGYGGGT	7	0
TAS2 forward	TTKRAWYGCRAGTTGCG	TAS3 reverse	CCGCTTAKTKATATGC	4	75
		TAS4 reverse	TATGCTTAARCTCRGCGGG	4	75
		TAS5 reverse	CTCCGCTTAKTKATATGC	117	44
TAS3 forward	TTKRAWYGCRAGTTGCGCC	TAS3 reverse	CCGCTTAKTKATATGC	4	75
		TAS4 reverse	TATGCTTAARCTCRGCGGG	4	75
		TAS5 reverse	CTCCGCTTAKTKATATGC	4	75
UniPlantF	TGTGAATTGCARRATYCMG	UniPlantR	CCCGHYTGAYYTGRGGTCDC	33	100
		Seed2 reverse	ATATGCTTAAAYTCAGCGGGYV	3	0
Seed2 forward	TTTGAACGCAMRTTGCGCC	Seed2 reverse	ATATGCTTAAAYTCAGCGGGYV	3	67
		UniPlantR	CCCGHYTGAYYTGRGGTCDC	3	67

Supplementary Table S3a. Mauritian species (native and exotic) used for primer design, alongside Order, Family and local name (where present). All Genbank accession numbers (A/N) are from sequences uploaded from a separate study[1]

Order	Family	Species	Local name	GenBank A/N
Apiales	Araliaceae	*Polyscias maraisiana*	Bois de Boeuf, Bois d'éponge	KY700450
Arecales	Arecaceae	*Hyophorbe lagenicaulis*	Palmiste Bouteille, Palmiste gargoulett	KY700379
Asparagales	Asparagaceae	*Asparagus setaceus*	Liane asperge	KY700230
Asparagales	Asparagaceae	*Asparagus umbellatus*	Asperge sauvage	KY700233
Asparagales	Orchidaceae	*Oeoniella polystachys*	-	KY700424
Asparagales	Xanthorrhoeaceae	*Aloe tormentorii*	Mazambron	KX689270
Asterales	Asteraceae	*Chromolaena odorata*	-	KY700271
Asterales	Asteraceae	*Psiadia arguta*	Baume de l'île Plate	KY700461
Asterales	Goodeniaceae	*Scaevola taccada*	Veloutier vert	KY700472
Boraginales	Boraginaceae	*Cordia curassavica*	Herbe Condé	KY700286
Boraginales	Boraginaceae	*Hilsenbergia petiolaris*	Bois pipe	KY700373
Boraginales	Boraginaceae	*Tournefortia argentea*	Veloutier blanc	KY700514
Caryophyllales	Amaranthaceae	*Aerva congesta*	-	KY700209
Caryophyllales	Amaranthaceae	*Amaranthus dubius*	Brède malabar	KY700217
Caryophyllales	Petiveriaceae	*Rivina humilis*	Petite groseille	KY700467
Caryophyllales	Portulacaceae	*Portulaca oleracea*	Pourpier rouge	KY700453
Celastrales	Celastraceae	*Cassine orientalis*	Bois d'olive	KY700255
Celastrales	Celastraceae	*Maytenus pyria*	Bois à poudre	KY700412
Ericales	Ebenaceae	*Diospyros tesselaria*	Bois d'ébène noir	KY700307
Ericales	Lecythidaceae	*Foetidia mauritiana*	Bois puant	KY700361
Ericales	Sapotaceae	*Sideroxylon boutonianum*	Bois de fer	KX689341
Fabales	Fabaceae	*Caesalpinia bonduc*	Cadoque	KY700251
Fabales	Fabaceae	*Dendrolobium umbellatum*	Bois malgache	KX689290

Order	Family	Species	Local name	GenBank A/N
Fabales	Fabaceae	*Desmanthus virgatus*	Petit acacia	KY700299
Fabales	Fabaceae	*Gagnebina pterocarpa*	Acacia indigene	KY700363
Fabales	Fabaceae	*Leucaena leucocephala*	Acacia indigène	KY700392
Fabales	Fabaceae	*Millettia pinnata*	Pongame	KY700415
Fabales	Fabaceae	*Pithecellobium dulce*	Cassie de Manille	KY700366
Fabales	Fabaceae	*Sophora tomentosa*	Bois chapelet	KY700495
Gentianales	Apocynaceae	*Catharanthus roseus*	Pervenche de Madagascar	KY700261
Gentianales	Apocynaceae	*Cynanchum staubii*	Liane calle	KX689283
Gentianales	Apocynaceae	*Ochrosia borbonica*	Bois jaune	KX689310
Gentianales	Apocynaceae	*Secamone dilapidens*	Liane bois d'olive, liane a ouate	KX689337
Gentianales	Apocynaceae	*Tylophora coriacea*	Ipéca du Pays	KY700526
Gentianales	Rubiaceae	*Coffea myrtifolia*	-	KY700288
Gentianales	Rubiaceae	*Coptosperma borbonica*	Bois de rat	KY700282
Gentianales	Rubiaceae	*Fernelia buxifolia*	Bois buis	KY700341
Gentianales	Rubiaceae	*Morinda citrifolia*	Bois tortue	KY700418
Gentianales	Rubiaceae	*Oldenlandia sieberi*	-	KX689313
Lamiales	Acanthaceae	*Asystasia gangetica*	Herbe pistache	KY700228
Lamiales	Acanthaceae	*Barleria observatrix*	-	KX689273
Lamiales	Bignoniaceae	*Tabebuia pallida*	Técoma	KX689347
Lamiales	Lamiaceae	*Premna serratifolia*	Bois sureau	KY700459
Lamiales	Lantaneae	*Lantana camara*	Vieille fille	KY700389
Lamiales	Lauraceae	*Clerodendrum heterophyllum*	Bois cabris	KY700274
Lamiales	Oleaceae	*Chionanthus ayresii*	Bois blanc	KX689274
Lamiales	Oleaceae	*Olea europaea var. africana*	Olivier de bourbon	KY700425
Malpighiales	Erythroxylaceae	*Erythroxylum sideroxyloides*	Bois de ronde	KY700318
Malpighiales	Euphorbiaceae	*Acalypha indica*	Herbe chatte	KY700205

Order	Family	Species	Local name	GenBank A/N
Malpighiales	Euphorbiaceae	*Euphorbia hirta*	Jean Robert	KY700326
Malpighiales	Euphorbiaceae	*Euphorbia prostrata*	Rougette	KY700340
Malpighiales	Euphorbiaceae	*Stillingia lineata*	Fangame	KY700505
Malpighiales	Passifloraceae	*Passiflora suberosa*	Liane poc poc	KY700430
Malpighiales	Passifloraceae	*Turnera angustifolia*	-	KX689353
Malpighiales	Phyllanthaceae	*Margaritaria anomala*	Bois chenille	KY700409
Malpighiales	Phyllanthaceae	*Phyllanthus casticum*	Bois castique	KY700442
Malpighiales	Phyllanthaceae	*Phyllanthus mauritianus*	-	KX689319
Malpighiales	Phyllanthaceae	*Phyllanthus revaughanii*	-	KX689324
Malpighiales	Phyllanthaceae	*Phyllanthus tenellus*	-	KY700446
Malpighiales	Salicaceae	*Flacourtia indica*	Prune malgache	KY700356
Malpighiales	Salicaceae	*Ludia mauritiana*	Bois mozambique	KY700403
Malvales	Malvaceae	*Dombeya mauritiana*	-	KY700311
Malvales	Malvaceae	*Hibiscus tiliaceus*	Var	KY700376
Malvales	Malvaceae	*Trochetia boutoniana*	-	KY700517
Malvales	Malvaceae	*Urena lobata*	Herbe panier	KY700528
Malvales	Thymelaeaceae	*Wikstroemia indica*	Herbe tourterelle	KY700531
Myrtales	Combretaceae	*Terminalia bentzoe*	Bois benjoin	KX689350
Myrtales	Lythraceae	*Pemphis acidula*	Bois matelot	KY700436
Myrtales	Myrtaceae	*Eugenia lucida*	Bois clou	KY700332
Oxalidales	Oxalidaceae	*Oxalis corniculata*	Petite oseille	KY700428
Poales	Cyperaceae	*Cyperus dubius*	-	KY700386
Poales	Cyperaceae	*Fimbristylis cymosa*	-	KY700346
Poales	Poaceae	*Vetiveria arguta*	-	KX689356
Rosales	Moraceae	*Ficus reflexa*	Lafouche bâtard	KY700354
Rosales	Moraceae	*Ficus rubra*	Affouche rouge	KX689294

Order	Family	Species	Local name	GenBank A/N
Rosales	Rhamnaceae	*Scutia myrtina*	Liane bambara	KY700477
Santalales	Santalaceae	*Santalum album*	Bois de santal	KY700470
Sapindales	Anacardiaceae	*Poupartia borbonica*	Bois poupart	KX689316
Sapindales	Burseraceae	*Protium obtusifolium*	Colophane bâtard	KY700457
Sapindales	Meliaceae	*Turraea thouarsiana*	Bois quivi	KY700524
Sapindales	Sapindaceae	*Dodonaea viscosa*	Bois de reinette	KY700314
Solanales	Convolvulaceae	*Ipomoea violacea*	-	KX689306
Solanales	Convolvulaceae	*Ipomoea obscura*	-	KY700384
Vitales	Vitaceae	*Cyphostemma mappia*	Mapou	KY700293

Supplementary Table S3b. UK species used for primer design, along with Order, Family and common name. Accession numbers beginning KT9486 are those uploaded from this study, the rest were downloaded from GenBank. All species were either known from previous studies of turtle dove diet[2,3], or common at our field sites or in supplementary or planted seed mixes[4] . Species with (spp.) after the common name are those which were not identified to the species level in previous dietary studies and for which we selected a representative species for primer design. Where multiple accession numbers are provided, these sequences were stitched together in order to cover the entire ITS2 and primer binding regions.

Order	Family	Species	Common Name	Genbank accession number (s)
Apiales	Apiaceae	*Anthriscus sylvestris*+	Cow parsley	AY548228 and KT948614
Asterales	Asteraceae	*Anthemis cotula*	Stinking chamomile	EU179216
Asterales	Asteraceae	*Carthamus tinctorius*+	Safflower	JQ230977 and KT948630
Asterales	Asteraceae	*Cirsium vulgare*	Spear thistle (spp.)	JX867638
Asterales	Asteraceae	*Guizotia abyssinica*+^	Niger seed	KT948615
Asterales	Asteraceae	*Helianthus annuus*+	Sunflower	JN115024
Asterales	Asteraceae	*Helminthotheca echoides*	Bristly ox-tongue	AF528491
Asterales	Asteraceae	*Senecio vulgaris*+	Groundsel	EF538396 and KT948631
Brassicales	Brassicaceae	*Brassica napus*+	Oil seed rape	JQ085860 and KT948616
Brassicales	Brassicaceae	*Capsella bursa-pastoris*+	Shepherd’s purse	DQ310531 and KT948632
Brassicales	Brassicaceae	*Sinapsis alba*	Field mustard	FJ609733
Brassicales	Resedaceae	*Reseda lutea*^	Wild mignonette	DQ987096*
Caryophyllales	Amaranthaceae	*Atriplex patula*	Orache	HM005859*
Caryophyllales	Caryophyllaceae	*Cerastium fontanum*	Common mouse-ear	GU444015
Caryophyllales	Caryophyllaceae	*Silene latifolia subsp. alba*	White campion	AY594308
Caryophyllales	Caryophyllaceae	*Silene vulgaris*	Bladder campion	FN821149
Caryophyllales	Caryophyllaceae	*Spergula arvensis*	Corn spurrey	JX274532

Order	Family	Species	Common Name	Genbank accession number (s)
Caryophyllales	Caryophyllaceae	*Stellaria graminea*	Lesser stitchwort (spp.)	AY594304
Caryophyllales	Caryophyllaceae	*Stellaria media*[+]	Chickweed	JN589063 and KT948633
Caryophyllales	Chenopodiaceae	*Chenopodium album*[+]	Fat hen	FN561552 and KT948617
Caryophyllales	Polygonaceae	*Persicaria maculosa*[+]	Redshank	HQ843137 and KT948635
Caryophyllales	Polygonaceae	*Polygonum aviculare*[+]	Knotgrass	KJ025070
Caryophyllales	Polygonaceae	*Rumex obtusifolius*[+]	Broad-leaved dock	GQ340059*
Ericales	Primulaceae	*Anagallis arvensis*[+]	Scarlet pimpernel	AY855135 and KT948628
Fabales	Fabaceae	*Lotus corniculatus*[+]	Birds-foot trefoil	DQ312207 and KT948621
Fabales	Fabaceae	*Medicago lupulina*[+]	Black medick	DQ311980
Fabales	Fabaceae	*Trifolium pratense*[+]	Red clover	AF053171 and KT948619
Fabales	Fabaceae	*Trifolium repens*[+]	White clover	DQ311962 and KT948620
Fabales	Fabaceae	*Vicia sativa*[+]	Common vetch	KJ787165
Gentianales	Rubiaceae	*Galium aparine*[+]	Goosegrass	DQ006036
Geraniales	Geraniaceae	*Geranium dissectum*[+]	Cut-leaved cranesbill	AY944413 and KT948622
Lamiales	Plantaginaceae	*Veronica persica*[+]	Common field speedwell	AF313001 and KT948624
Lamiales	Scrophulariaceae	*Kickxia spuria*	Round-leaf fluellen	AF513880
Malpighiales	Euphorbiaceae	*Euphorbia esula*	Green spurge (spp.)	JN010042
Malpighiales	Violaceae	*Viola arvensis*[+]	Field pansy	DQ005347 and KT948636
Malpighiales	Violaceae	*Viola tricolor*	Heartsease	DQ055406
Poales	Poaceae	*Alopecurus myosuroides*[+^]	Black grass	KT948627
Poales	Poaceae	*Festuca pratensis*	Meadow fescue (spp.)	KJ598995
Poales	Poaceae	*Hordeum vulgare*[+]	Barley	KM217265 and KT948626
Poales	Poaceae	*Panicum miliaceum*[+]	Millet	KT948629 and JX576677
Poales	Poaceae	*Poa annua*[+]	Meadow grass	KJ599003 and KT948634
Poales	Poaceae	*Poa trivialis*	Rough meadow-grass (spp.)	KJ598983

Order	Family	Species	Common Name	Genbank accession number (s)
Poales	Poaceae	*Sorghum bicolor*[+]	White sorghum	GQ856358
Poales	Poaceae	*Triticum aestivum*[+]	Wheat	KF482086 and KT948625
Poales	Poaceae	*Zea mays*[+]	Maize	DQ683016*
Ranunculales	Papaveraceae	*Fumaria officinalis*[+]	Common fumitory	HE603306 and KT948623
Ranunculales	Papaveraceae	*Papaver rhoeas*	Poppy	DQ912886
Ranunculales	Ranunculaceae	*Ranunculus repens*	Creeping buttercup	JN115047*
Rosales	Urticaceae	*Urtica dioica*	Common nettle	KF454275 and KF137936
Solanales	Convolvulaceae	*Convolvulus arvensis*[+]	Field bindweed	AY558826

^Sequence does not or only partially overlaps forward primer region

* Sequence does not or only partially overlaps reverse primer region

Supplementary Table S4. Results of *in vitro* mock community experiment. PCR mix treatments: (i) Equal: equal proportion of plants with short and long UniPlant amplicons (3 long and 3 short species); (ii) Short Bias: PCR mix favoured short amplicon species (2 long and 4 short species); Long Bias: PCR mix favoured long amplicon species (4 long and 2 short species). The DNA concentration in each PCR mix was determined by high-resolution capillary electrophoresis using a QIAxcel (Qiagen, Manchester, UK). *Amplicon lengths determined by a QIAxcel include primers and so differ from those specified in the main text.

Treatment	PCR mix	Amplicon length determine by QIAxcel (bp)*	Long or short amplicon	DNA concentration (ng/uL)
Equal	1	301	short	13.96
		415	long	33.06
	2	298	short	27.3
		401	long	15.25
	3	301	short	26.48
		363	long	25.26
	4	297	short	29.02
		389	long	10.91
	5	299	short	25.43
		413	long	10.12
Short Bias	6	298	short	34.57
		380	long	11.79
	7	297	short	31
		420	long	13.46
	8	299	short	29.07
		368	long	9.99
	9	298	short	21.53
		363	long	27.88
	10	298	short	35.61
		388	long	8.41

Treatment	PCR mix	Amplicon length determine by QIAxcel (bp)*	Long or short amplicon	DNA concentration (ng/uL)
Long Bias	11	296	short	20.68
		368	long	23.59
	12	304	short	21.49
		366	long	30.33
	13	301	short	9.2
		404	long	34.17
	14	298	short	12.01
		403	long	41.52
	15	296	short	18.35
		395	long	18.34

Supplementary Table S5a. Species amplified and sequenced on an Illumina MiSeq platform using UniPlantF and UniPlantR from faecal samples from Telfair skink (n=246). Those taxa that could be identified to genus only are identified in bold. Those taxa identified based on matches on GenBank alone as opposed to a comprehensive DNA barcode library are marked with *.

Order	Family	Species	Number of samples
Apiales	Araliaceae	*Polyscias maraisiana*	16
Asparagales	Asparagaceae	*Asparagus setaceus*	7
Asparagales	Asparagaceae	*Asparagus umbellatus*	3
Asterales	Asteraceae	*Tridax procumbens*	3
Asterales	Asteraceae	*Bidens pilosa*	1
Asterales	Goodeniaceae	*Scaevola taccada*	19
Boraginales	Boraginaceae	*Hilsenbergia petiolaris*	94
Brassicales	Caricaceae	*Carica papaya*	1
Caryophyllales	Aizoaceae	*Sesuvium ayresii*	1
Caryophyllales	Amaranthaceae	*Achyranthes aspera*	2
Caryophyllales	Petiveriaceae	*Rivina humilis*	5
Caryophyllales	Portulacaceae	*Portulaca oleracea*	3
Celastrales	Celastraceae	*Cassine orientalis*	3
Celastrales	Celastraceae	*Maytenus pyria*	5
Ericales	Ebenaceae	*Diospyros tesselaria*	1
Ericales	Ebenaceae	*Diospyros egrettarum*	7
Fabales	Fabaceae	*Caesalpinia bonduc*	5
Fabales	Fabaceae	*Desmanthus virgatus*	1
Fabales	Fabaceae	*Millettia pinnata*	1
Fabales	Fabaceae	*Gagnebina pterocarpa*	6
Fabales	Fabaceae	*Leucaena leucocephala*	78
Fabales	Fabaceae	*Pithecellobium dulce*	8

Order	Family	Species	Number of samples
Fabales	Fabaceae	*Rhynchosia viscosa*	1
Gentianales	Apocynaceae	*Cynanchum staubii*	7
Gentianales	Apocynaceae	*Tylophora coriacea*	7
Gentianales	Rubiaceae	*Morinda citrifolia*	44
Lamiales	Acanthaceae	*Asystasia gangetica*	12
Lamiales	Lamiaceae	*Premna serratifolia*	42
Lamiales	Lauraceae	*Clerodendrum heterophyllum*	7
Lamiales	Plantaginaceae	***Plantago sp.****	4
Lamiales	Verbenaceae	*Stachytarpheta jamaicensis*	13
Malpighiales	Euphorbiaceae	*Acalypha indica*	3
Malpighiales	Euphorbiaceae	*Euphorbia hirta*	19
Malpighiales	Euphorbiaceae	*Euphorbia thymifolia*	2
Malpighiales	Passifloraceae	*Passiflora suberosa*	108
Malpighiales	Passifloraceae	*Turnera angustifolia*	7
Malpighiales	Phyllanthaceae	*Phyllanthus amarus*	1
Malpighiales	Phyllanthaceae	*Phyllanthus casticum*	5
Malpighiales	Phyllanthaceae	*Phyllanthus mauritianus*	1
Malpighiales	Phyllanthaceae	*Margaritaria anomala*	89
Malpighiales	Salicaceae	*Flacourtia indica*	4
Malvales	Malvaceae	*Abutilon indicum*	2
Malvales	Malvaceae	*Hibiscus tiliaceus*	44
Malvales	Malvaceae	*Thespesia populnea*	29
Malvales	Malvaceae	*Sida pusila*	3
Malvales	Thymelaeaceae	*Wikstroemia indica*	5
Myrtales	Combretaceae	*Terminalia bentzoe*	1
Myrtales	Myrtaceae	*Eugenia lucida*	37

Order	Family	Species	Number of samples
Poales	Cyperaceae	*Cyperus dubius*	40
Poales	Poaceae	*Cenchrus echinatus*	4
Poales	Poaceae	*Dactyloctenium ctenoides*	1
Poales	Poaceae	*Digitaria horizontalis*	8
Poales	Poaceae	*Eragrostis amabilis*	34
Poales	Poaceae	*Stenotaphrum dimidiatum*	6
Poales	Poaceae	***Triticum* sp.***	**5**
Poales	Poaceae	*Chloris barbata*	3
Poales	Poaceae	***Holcus* sp.***	**3**
Poales	Poaceae	***Agrostis* sp.***	**1**
Poales	Poaceae	*Poa trivialis**	1
Poales	Poaceae	*Zea mays**	3
Polypodiales	Polypodiaceae	*Phymatodes scolopendria*	2
Rosales	Moraceae	*Ficus reflexa*	142
Rosales	Moraceae	*Ficus rubra*	183
Rosales	Rhamnaceae	*Gouania tiliifolia*	7
Rosales	Rhamnaceae	*Scutia myrtina*	10
Rosales	Rhamnaceae	*Colubrina asiatica*	1
Santalales	Santalaceae	*Santalum album*	11
Sapindales	Anacardiaceae	*Coptosperma borbonica*	7
Sapindales	Anacardiaceae	*Poupartia borbonica*	6
Sapindales	Meliaceae	*Turraea thouarsiana*	3
Sapindales	Sapindaceae	*Dodonaea viscosa*	5
Solanales	Convolvulaceae	*Dichondra repens*	1
Solanales	Convolvulaceae	*Ipomoea violacea*	130
Solanales	Convolvulaceae	*Ipomoea obscura*	9

Order	Family	Species	Number of samples
Solanales	Solanaceae	*Nicotiana tabacum*	2
Solanales	Solanaceae	*Solanum americanum*	25

Supplementary Table S5b. Species amplified and sequenced on an Illumina MiSeq platform using UniPlantF and UniPlantR from faecal samples from Stock doves (n=13). Those taxa that could be identified to genus only are identified in bold. 7.62 ± 0.94 taxonomic units were identified per faecal sample, with a total of 40 taxonomic units identified from 13 faecal samples.

Order	Family	Species	Number of samples
Apiales	Apiaceae	***Anthriscus* sp.**	1
Apiales	Apiaceae	*Pastinaca sativa*	1
Asterales	Asteraceae	*Cirsium vulgare*	1
Asterales	Asteraceae	*Guizotia abyssinica*	2
Asterales	Asteraceae	*Helianthus annuus*	1
Asterales	Asteraceae	*Lapsana communis*	1
Boraginales	Boraginaceae	*Borago officinalis*	8
Boraginales	Boraginaceae	***Symphytum* sp.**	1
Brassicales	Brassicaceae	*Brassica napus*	5
Brassicales	Brassicaceae	*Brassica oleracea*	1
Brassicales	Brassicaceae	***Brassica* sp.**	8
Brassicales	Brassicaceae	Brassicaceae	6
Brassicales	Brassicaceae	*Thlaspi arvense*	1
Brassicales	Resedaceae	*Reseda lutea*	2
Caryophyllales	Amaranthaceae	***Atriplex* sp.**	2
Caryophyllales	Amaranthaceae	*Chenopodium polyspermum*	1
Caryophyllales	Caryophyllaceae	*Stellaria media*	1
Ericales	Primulaceae	*Anagallis arvensis*	11
Ericales	Primulaceae	Primulaceae	1
Fabales	Fabaceae	*Vicia sativa*	1
Fagales	Fagaceae	***Quercus* sp.**	1

Order	Family	Species	Number of samples
Geraniales	Geraniaceae	*Geranium dissectum*	4
Geraniales	Geraniaceae	*Geranium lucidum*	1
Lamiales	Plantaginaceae	*Plantago lanceolata*	1
Malpighiales	Violaceae	*Viola arvensis*	1
Poales	Poaceae	*Dactylis glomerata*	4
Poales	Poaceae	***Hordeum* sp.**	1
Poales	Poaceae	***Lolium* sp.**	1
Poales	Poaceae	*Panicum miliaceum*	8
Poales	Poaceae	*Poa annua*	1
Poales	Poaceae	*Poa infirma*	1
Poales	Poaceae	***Poa* sp.**	2
Poales	Poaceae	Poaceae	5
Poales	Poaceae	***Triticum* sp.**	2
Ranunculales	Ranunculaceae	*Clematis vitalba*	1
Rosales	Cannabaceae	*Cannabis sativa*	1
Rosales	Rosaceae	***Prunus* sp.**	1
Rosales	Rosaceae	Rosaceae	1
Rosales	Rosaceae	***Rubus* sp.**	4
Rosales	Urticaceae	*Urtica dioica*	2

Supplementary Figure S1. Plot displaying the results of a mock community experiment to explore DNA fragment size bias at the PCR stage. Blue lines are predicted values generated by a generalised linear mixed effects model created using the *lme4* package[5] in R[6]. The treatment names are listed in orange boxes, long or short amplicons are labelled on the x-axis and DNA concentration forms the y-axis. The plot was created using the R package *visreg*[7]

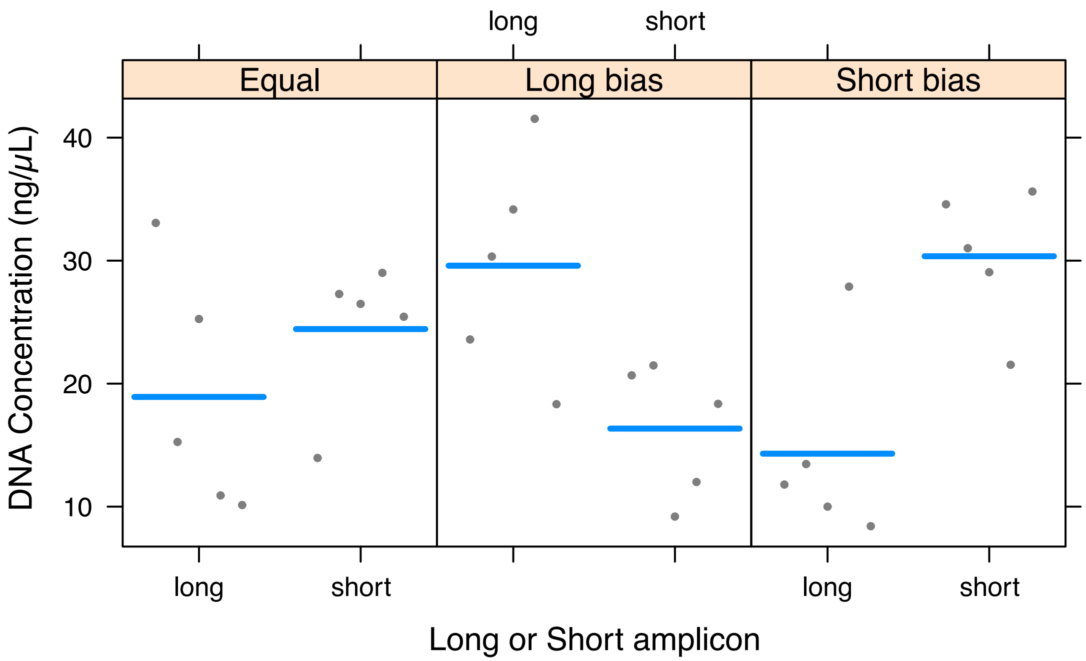

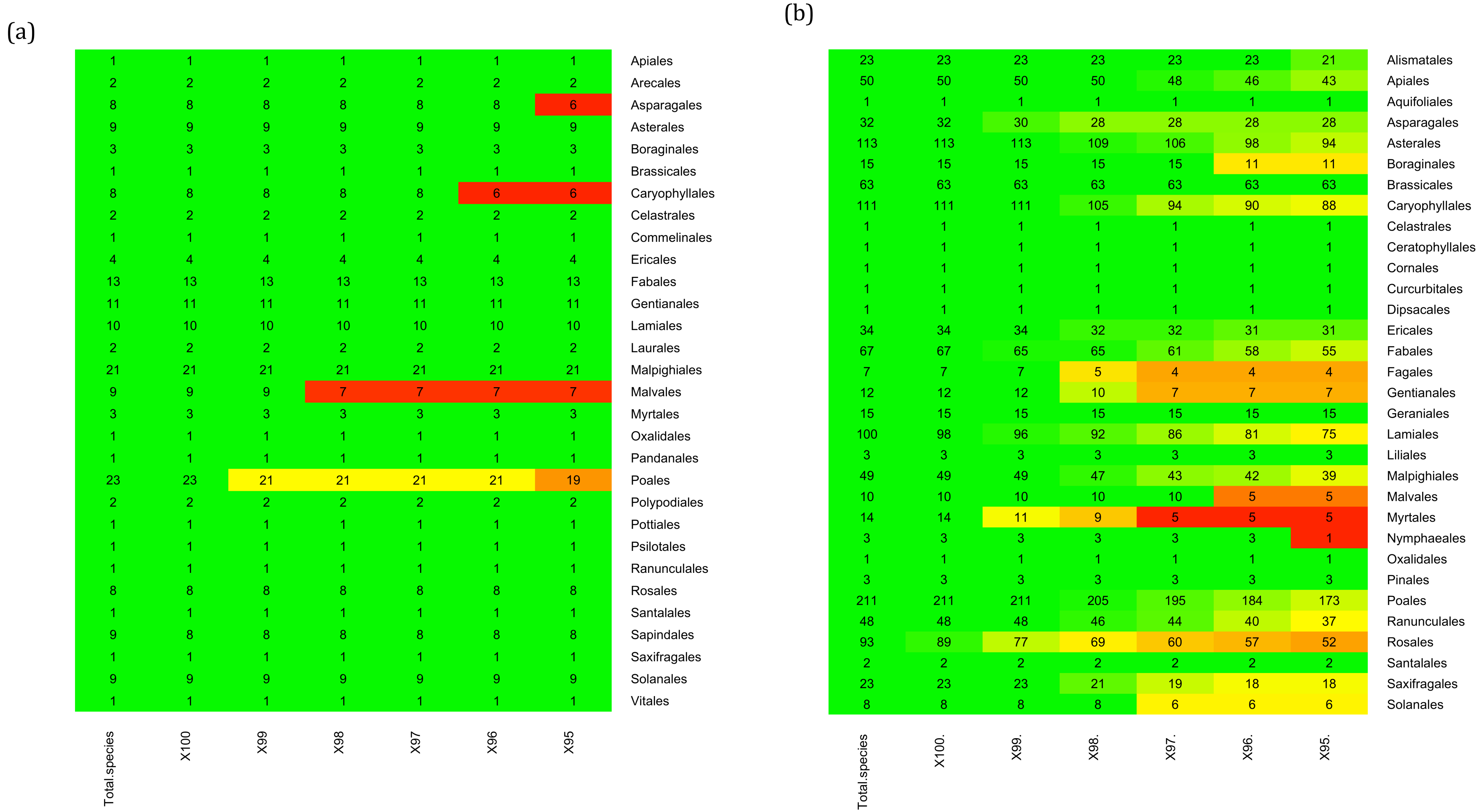

Supplementary Figure S2. Order-level summary of clustering thresholds for the full UniPlant amplicon between 95 and 100% for (a) Mauritian, n=167 species, and (b) UK databases, n=1116 species. Order names are listed on the y-axis and clustering threshold forms the x-axis. The colour of the cells represents the percentage of species within an order that can be identified to species level at a given clustering threshold. Colour gradient from green through to red signifies high species-level resolution moving towards poor species-level resolution.

Supplementary Note S1. Methods for sample collection and downstream data analysis of NGS data from UK doves and pigeons (specifically stock doves *Columba oenas*, whose dietary data are presented here) and Mauritian birds and reptiles (specifically the Telfair's skink *Leiolopisma telfairii*, whose dietary data are presented here), along with results from their corresponding NGS runs. This information is also provided in Dunn *et al.*[8] and Moorhouse-Gann[1].

Sample collection

Faecal samples were collected from adult and nestling stock doves as part of a 4-year autecological study of turtle dove breeding ecology at 12 farmland sites across Essex, Suffolk, Cambridgeshire and Norfolk, UK[4]. Stock dove faecal samples were collected at four of these sites, in Essex and Suffolk, near Great Wigborough (51°79'N, 0°85'W), Great Ashfield (52°27'N, 0°92'W), Mark's Tey (51°87'N, 0°76'W) and Stow Maries (51°66'N, 0°65'W).

Adult doves were caught using whoosh and mist nets[9] at temporarily-baited sites in areas either where birds had previously been seen feeding, or where farmers provided grain, during May, June and July 2011–2014. When caught, birds were weighed and maximum wing chord measured[9]. We collected 10 faecal samples from adult stock doves, either directly from the bird, or from the inside of clean bird bags within which the birds were temporarily held after capture. All faecal samples were frozen at -20°C as soon as possible after collection (1–8 hrs) until subsequent analysis. We also collected faecal samples from 3 stock dove nestlings that were opportunistically ringed when encountered in nestboxes as the correct developmental stage. Faecal samples from nestlings were collected between June – September, 2011–2014.

Faecal samples were collected from Telfair's skinks between July 2014 and June 2015 as part of a study examining trophic interactions on Mauritian islands[1].

Skinks were caught by hand or noose on Ile aux Aigrettes (20°42'S, 57°73'E), Mauritius. Each individual's unique passive integrated transponder (PIT) tag number was read, morphometric information recorded and an abdominal massage applied to induce defecation. A total of 274 faecal samples were collected from adult Telfair's skinks and subsequently stored in polythene bags with silica gel crystals.

Faecal analysis

DNA was extracted from approximately 200 mg of each stock dove faecal sample described here, and samples from adults, nestlings and nests (post-fledging) of three other dove and pigeon species (total sample n = 121) using a QIAamp DNA Stool Mini Kit (Qiagen,

Manchester, UK) with slight modifications to the manufacturer's instructions detailed in Dunn *et al.*[10], using negative extraction controls (n=6) throughout. We used primers UniPlantF and UniPlantR to amplify a 187–380 bp region encompassing the ITS2 region of plant nuclear DNA and labelled each sample with a unique combination of forward and reverse MID tags[11]. PCRs were carried out in 10 µl reaction volumes containing 5 µl multiplex buffer (Qiagen, Manchester, UK), 2.6 µl H_2O, 0.2 µl each primer (10 mM) and 2 µl DNA. Reaction conditions were initial denaturation at 95°C for 15 minutes, 40 cycles of 95°C for 30 s, 56°C for 30 s, 72°C for 1 min followed by a final extension of 72°C for 10 min, and all PCR reactions were carried out on a Gene Amp® PCR System 9700 (Applied Biosystems, Foster City, CA). Samples were pooled according to intensity of the PCR product on a 1% agarose gel stained with SYBR®Safe (ThermoFisher Scientific, Paisley, UK) when compared to a standardised 100 bp ladder and subsequently quantified using a BioAnalyzer (Agilent Technologies, Santa Clara, CA) to check peak amplicon size and DNA concentration. Only samples where a clear band was visible following electrophoresis were processed further. Samples were purified in pools of similar DNA concentration using a QIAquick PCR Purification kit (Qiagen, Manchester, UK), quantified using a Qubit (ThermoFisher Scientific, Waltham, MA) and pools subsequently combined in order to provide an approximately equal amount of amplicon DNA from each faecal sample.

The pool of individually-tagged amplicons was used to prepare a library for paired end sequencing using the NEBNext Ultra DNA Library Prep Kit for Illumina (New England Biolabs, Ipswich, MA). The library was sequenced using 250 bp paired-end reads on a MiSeq desktop sequencer (Illumina, San Diego, CA).

In the Mauritian study, DNA from 36 – 44 mg of the dried Telfair's skink faecal samples described above was extracted using the QIAamp DNA Stool Mini Kit (Qiagen, Manchester, UK), with the following modifications: (i) faecal material was ground using a MP FastPrep®-24 at 5.5 m/s for 20 seconds; (ii) samples were vortexed with buffer ASL for 30 minutes; (ii) samples were vortexed with the InhibitEX tablet for 5 minutes, and subsequently centrifuged for 6 minutes; (iv) samples were incubated for 30 minutes with buffer AL at 73˚C; (v) DNA elution was carried out twice with 100 μ buffer AE after incubating at room temperature for 10 minutes. At least one DNA extraction negative was included in each DNA extraction session. The UniPlant primers and MID tags were applied as described above. PCRs were carried out in 20 µL reaction volumes using the same ratio of reagents as described above except with H_2O partially substituted for 2 µL of Q solution (Qiagen, Manchester, UK). Reaction conditions were initial denaturation at 95°C for 15 minutes, 40 cycles of 95°C for 30 s, 56°C for 90 s, 72°C for 90 s followed by a final extension of 72°C for 10 min. Samples in each PCR reaction were pooled according to intensity of the PCR product on a 2% agarose gel (as above) after quantifying the PCR product in a subset of samples from each gel, to verify band intensity, using the broad range assay with a Qubit Flourometer (ThermoFisher Scientific, Paisley, UK)..Only those samples with the lowest DNA concentrations (<15 ng/µL) were purified using a QIAquick PCR purification kit (Qiagen,

Manchester, UK). Here, more initial product was applied than final elute to simultaneously concentrate the purified PCR product. The DNA concentration in each PCR reaction pool was quantified once more before the concentration across all pools was equalised and all pools were pooled into one of two final pools: one for each Illumina Miseq run. Each final pool was run on an Agilent 2200 TapeStation with a D1000 ScreenTape (Agilent Technologies, Waldbronn), which indicated that there was insignificant primer dimer so no further purification steps were required. The final two pools of individually tagged amplicons were used for library preparation and sequencing, as described above.

Identification of plant species

For both stock dove and Telfair's skink datasets, paired-end Illumina sequences were filtered for quality using Trimmomatic v0.32[12] with a minimum quality score of 20 over a sliding window of 4 bp, retaining sequences with a minimum length of 135 bp. These were aligned using FLASH[13] and demultiplexed into faecal sample-specific files using the MID tag sequence with the "trim_seqs" command in Mothur[14], which also removes the MID and primer sequences from the reads. We then used the "derep_fulllength" and "uchime2_denovo" commands in the USEARCH software v9.2.64[15] to remove any sequences with fewer than 10 copies within a faecal sample and any potential chimeric sequences. For the skink dataset only, ITS2 sequences were extracted from all reads using ITSx[16] and USEARCH[15] was used once again to extract all unique ITS2 sequences. Analysis of species discrimination at the ITS2 region (this manuscript) suggests this region to be unsuitable for an approach of clustering similar sequences into molecular operational taxonomic units (MOTUs) due to the loss of ability to distinguish between species prior to the grouping of multiple polymorphisms within some plant species. Therefore, for both datasets we adopted a closest matching sequence approach to identify species within our samples [e.g.17, 20].

We took a sequence read-number approach to deal with any background contamination. First, for the stock dove dataset only, we examined sequences found only in samples with unused MID combinations (n=20) as these could only be attributed to background contaminants or "tag jumping"[18,19]. The highest number of reads for any of these sequences was 139, so we re-ran our initial de-replication step (using "derep_fulllength" in USEARCH) with this new sequence read threshold. We then assigned the resulting sequences to taxonomic unit using the BLAST algorithm[20] to search GenBank, combined with new sequences from our barcode library (GenBank accession numbers KT948614–KT948638). If a sequence had the smallest e-value matching only one species on GenBank, with >99% sequence identity, we assigned the sequence to that species[17]. If the sequence matched more than one species from the same genus, tribe or family (with a % match between 90 and 99), we assigned the sequence to the lowest common taxonomic unit up to the family level. sequence with <90% match to the closest matching species on GenBank, or for which BLAST returned no significant match

was discarded, as was any sequence for which the closest match included a bacterium or fungus. Next, for both datasets, we examined each unique sequence found in a negative sample, including unused MID combinations, PCR negatives (Stock dove n=2, Telfair's skink n=7) and extraction negatives (Stock dove n=6, Telfair's skink n=9) to deal with any specific contaminants within our samples. For each sequence, we identified the highest read number within a negative sample and removed this sequence from any sample where the read number was below this threshold. For the Telfair's skink dataset only, the Blastn algorithm[20] was used in Blast+[21] for taxonomic assignment, comparing all sequences to a comprehensive DNA barcode library of the Ile aux Aigrettes flora[1]. Sequences were assigned to taxa based on BIT score (as in [17,22]): if the highest BIT score was reserved to a match with a single species then species-level identification was achieved and the same rule was applied to genus-level matches. If a sequence failed to match a plant in the barcode library, the blastn algorithm[20] was used, as above, to search for matches on NCBI GenBank. This final dataset was cleaned further by removing those species which are known not to grow on Ile aux Aigrettes, e.g. those plant taxa known to be present in the supplementary feed of other species. Finally, for both datasets, sequences within each taxonomic unit were combined.

NGS results

The Illumina runs resulted in 12,592,989, 9,683,014 and 15,902,919 paired reads for the stock dove dataset and first and second pools of the Telfair's skink datasets respectively. Following filtering out of adapters and low quality sequences, 10,138,058, 4,301,210 and 11,251,688 sequences remained. Once sequences had been paired, we were left with 9,921,248, 4,094,216 and 8,923,922 aligned sequences. For the Stock dove dataset only, after eliminating reads without an exact match to primer sequences and MID tags 6,105,478 sequences remained (mean± SE for samples: 42,917 ± 2,871; for negatives and unused tag combinations: 1,930 ± 382). Following dereplication, our initial file contained 12,608 unique sequences, but after raising our filtering threshold (to 139 reads per identical sequence) based on read numbers from sequences found only in samples from unused MID combinations in order to eliminate background contamination, we had 1,192 unique sequences. 80 sequences showed poor matches (<90% identity or no match in GenBank) to the closest matching sequence, 64 sequences were from bacteria or fungi and 5 sequences were found at their highest read numbers in negative samples: these were discarded, leaving 1,043 unique sequences assigned to 143 taxonomic units. For the Telfair's skink dataset only, after eliminating reads without a match to primer sequences and MID tags (allowing for a 1bp mismatch) 3,068,969 & 3,020,763 sequences remained.

Supplementary Data S1 List of Genbank accession numbers for the DNA sequences used for *in silico* analyses in this study

LC076491, LC076483, EU687533, AM920399-AM920403, KX165423-KX167996, KT948614-KT948638, KY700199-KY700571, KY700573-KY700576, KX689270-KX689363,AB000330.1, AB019948.1, AB022736.1, AB023983.1, AB032039.1, AB080562.1, AB088584.1, AB118124.1, AB120207.1, AB198348.1, AB248848.1, AB248857.1, AB261684.1, AB261687.1, AB292041.1, AB359790.1, AB359802.1, AB541095.1, AB683270.1, AB689040.1, AB851487.1, AB851493.1, AF009082.1, AF019790.1, AF019857.1, AF019873.1, AF031962.1, AF031964.1, AF037014.1, AF037624.1, AF040009.1, AF040063.1, AF040076.1, AF041343.1, AF041353.1, AF072485.1, AF077895.1, AF077900.1, AF077904.1, AF078032.1, AF088203.1, AF091952.1, AF115160.1, AF130839.1, AF136621.1, AF137539.1, AF158952.1, AF163401.1, AF163494.1, AF164001.1, AF165832.1, AF167196.1, AF169236.1, AF169757.1, AF183568.1, AF189730.1, AF209811.1, AF216544.1, AF218505.1, AF245429.1, AF245430.1, AF265281.1, AF272278.1, AF283487.1, AF301441.1, AF303026.1, AF313032.1, AF313035.1, AF318646.1, AF318715.1, AF336215.1, AF336371.1, AF351088.1, AF351121.1, AF358872.1, AF361301.1, AF367618.1, AF387520.1, AF401114.1, AF419000.1, AF422136.1, AF426378.1, AF448794.1, AF450226.1, AF450229.1, AF469682.1, AF478941.1, AF497647.1, AF497689.1, AF505631.1, AF513874.1, AF513875.1, AF513883.1, AF513888.1, AF517101.1, AF528453.1, AF528486.1, AF528490.1, AF528491.1, AF531080.1, AF540073.1, AF547727.1, AF551727.1, AJ011473.1, AJ011479.1, AJ222839.1, AJ251663.1, AJ304908.1, AJ310965.1, AJ310977.1, AJ310980.1, AJ347901.1, AJ347913.1, AJ420994.1, AJ427757.1, AJ438215.1, AJ491666.1, AJ491674.1, AJ511770.1, AJ536581.1, AJ539529.1, AJ548963.1, AJ548984.1, AJ550588.1, AJ579441.1, AJ580551.1, AJ580557.1, AJ626769.1, AJ633339.1, AJ633340.1, AJ633417.1, AJ633446.1, AJ633466.1, AJ633471.1, AJ633476.1, AJ744931.1, AJ746409.1, AJ862704.1, AJ868086.1, AM117024.1, AM267278.1, AM267287.1, AM287271.1, AM420677.1, AM503876.2, AM711744.1, AM711747.1, AM905721.1, AM905723.1, AM905724.1, AM905725.1, AM920396.1, AM943384.1, AY035750.1, AY049799.1, AY091574.1, AY092898.1, AY092900.1, AY092907.1, AY101281.1, AY146446.1, AY148270.1, AY148280.1, AY148284.1, AY176157.1, AY177603.1, AY179028.1, AY207370.1, AY236181.1, AY237921.1, AY254530.1, AY254531.1, AY254532.1, AY254541.1, AY254544.1, AY254545.1, AY263631.1, AY263679.1, AY265134.1, AY290016.1, AY290017.1, AY325280.1, AY328303.1, AY330707.1, AY331478.1, AY335961.1, AY335962.1, AY338946.1, AY341385.1, AY351379.1, AY351385.1, AY357769.1, AY357794.1, AY362767.1, AY380861.1, AY380868.1, AY438320.1, AY492098.1, AY492108.1, AY506652.1, AY508213.1, AY515398.1, AY524764.1, AY538636.1, AY548225.1, AY552528.1, AY554108.1, AY557232.1, AY558826.1, AY575439.1, AY581801.1, AY591277.1, AY594303.1, AY603257.1, AY616730.1, AY634778.1, AY635025.1, AY635034.1, AY665850.1, AY712663.1, AY722431.1, AY722459.1, AY722470.1, AY723256.1, AY731259.1, AY740898.1, AY764040.1, AY764073.1, AY787405.1, AY839340.1, AY839344.1, AY857969.1, AY858597.1, AY870357.1, AY880236.1, AY880318.1, AY918196.1, AY926320.1, AY936278.1, AY974174.1, AY988396.1, AY996232.1, DQ005670.1, DQ005963.1, DQ005983.1, DQ005989.1, DQ005990.1, DQ006009.1, DQ006021.1, DQ006032.1, DQ006036.1, DQ006273.1, DQ022894.1, DQ059409.1, DQ066495.1,

DQ074223.1, DQ092929.1, DQ180745.1, DQ184479.1, DQ217769.1, DQ224364.1, DQ249825.1, DQ249831.1, DQ249853.1, DQ250322.1, DQ276850.1, DQ277637.1, DQ304566.1, DQ304570.1, DQ310524.1, DQ310530.1, DQ310531.1, DQ311965.1, DQ311970.1, DQ312107.1, DQ314189.1, DQ336817.1, DQ336826.1, DQ336830.1, DQ336833.1, DQ340170.1, DQ340518.1, DQ354164.1, DQ354169.1, DQ357547.1, DQ385576.1, DQ435067.1, DQ444720.1, DQ451818.1, DQ467430.1, DQ467575.1, DQ468390.1, DQ518397.1, DQ539573.1, DQ539575.1, DQ539578.1, DQ539580.1, DQ539589.1, DQ539598.1, DQ539600.1, DQ642002.1, DQ667241.1, DQ667243.1, DQ667244.1, DQ667250.1, DQ667291.1, DQ667301.1, DQ683016.1, DQ888637.1, DQ912878.1, DQ912883.1, DQ975357.1, DQ987188.1, DQ996585.1, EF017402.1, EF057696.1, EF060391.1, EF065545.1, EF079387.1, EF090599.1, EF091578.1, EF127014.1, EF127040.1, EF142996.1, EF153087.1, EF185366.1, EF190030.1, EF195130.1, EF210971.1, EF368007.1, EF395535.1, EF397234.1, EF407926.1, EF416663.1, EF419452.1, EF427950.1, EF427951.1, EF436990.1, EF437224.1, EF494236.1, EF494737.1, EF517848.1, EF526367.1, EF526374.1, EF526385.1, EF538230.1, EF556350.1, EF560689.1, EF565132.1, EF577510.1, EF581935.1, EF635453.1, EF635458.1, EF635464.1, EF635471.1, EF660599.1, EU016360.1, EU070650.1, EU072550.1, EU102706.1, EU102747.1, EU179213.1, EU179215.1, EU179216.1, EU239681.1, EU239683.1, EU288566.1, EU307117.1, EU314901.1, EU331123.1, EU352241.1, EU352242.1, EU366281.1, EU391323.1, EU401308.1, EU592013.1, EU594911.1, EU606218.1, EU628253.1, EU655615.1, EU669091.1, EU687658.1, EU699446.1, EU707255.1, EU711228.1, EU720548.1, EU747245.1, EU785941.1, EU785983.1, EU792342.1, EU792364.1, EU812820.1, EU827106.1, EU850000.1, EU850027.1, EU850665.1, EU912092.1, FJ010604.2, FJ013226.1, FJ169534.1, FJ213869.1, FJ377658.1, FJ378588.1, FJ394658.1, FJ415110.1, FJ428646.1, FJ454870.1, FJ593180.1, FJ599757.1, FJ609733.1, FJ694190.1, FJ695466.1, FJ696962.1, FJ751757.1, FJ763189.1, FJ763192.1, FJ790040.1, FJ790048.1, FJ790050.1, FJ796906.1, FJ796919.1, FJ814654.1, FJ821024.1, FJ867401.1, FJ895960.1, FJ915103.2, FJ945920.1, FJ980316.1, FJ980324.1, FJ980347.1, FJ980407.1, FM243981.1, FM958516.1, FM995387.1, FM995395.1, FN263234.1, FN645827.1, FN645833.1, FN645882.1, FR715299.1, GQ285223.1, GQ285224.1, GQ285225.1, GQ285235.1, GQ373320.1, GQ379288.1, GQ395466.1, GQ464812.1, GQ464834.1, GQ464836.1, GQ464843.1, GQ470550.1, GQ470555.1, GQ478098.1, GQ478113.1, GQ856358.1, GQ862376.1, GQ888926.1, GQ901329.1, GU062347.1, GU067554.1, GU067564.1, GU176630.1, GU188570.1, GU217797.1, GU256780.1, GU323359.1, GU329698.1, GU350650.1, GU440883.1, GU444005.1, GU444008.1, GU444015.1, GU724280.1, GU724298.1, GU724302.1, GU818507.1, GU818542.1, GU818553.1, GU818558.1, GU818724.1, GU969621.1, GU977052.1, GU983033.1, HE586018.1, HE602438.1, HE602464.1, HE603307.1, HE603311.1, HE603315.1, HE603320.1, HF952973.1, HG315518.1, HG424173.1, HG518071.1, HG797486.1, HG797487.1, HG915798.1, HG934728.1, HM010981.1, HM049903.1, HM116949.1, HM176655.1, HM182015.1, HM193529.1, HM204888.1, HM235960.1, HM240422.1, HM357931.1, HM468330.1, HM468337.1, HM542614.1, HM587574.1, HM593905.1, HQ142591.1, HQ176475.1, HQ284120.1, HQ288850.1, HQ393700.1, HQ407556.1, HQ414209.1, HQ414215.1, HQ442261.1, HQ445941.1, HQ456394.1, HQ456398.1, HQ456401.1, HQ456403.1, HQ456420.1, HQ600516.1, HQ600554.1, HQ615081.1, HQ658361.1, HQ687165.1, HQ823435.1, HQ858880.1, HQ858884.1, HQ858911.1, HQ859001.1, HQ896628.1,

HQ917107.1, HQ993100.1, JF313179.1, JF331882.1, JF421553.1, JF780965.1, JF805747.1, JF900504.1, JF904803.1, JF907423.1, JF926362.1, JF976139.1, JF976297.1, JF976473.1, JF976634.1, JF977428.1, JF980331.1, JN009831.1, JN010036.1, JN113283.1, JN115012.1, JN115024.1, JN115053.1, JN234733.1, JN235076.1, JN235079.1, JN235087.1, JN235102.1, JN247411.1, JN315918.1, JN315931.1, JN375572.1, JN389222.1, JN400254.1, JN407499.1, JN407526.1, JN564914.1, JN575347.1, JN575417.1, JN578321.1, JN589051.1, JN589087.1, JN589095.1, JN589103.1, JN589107.1, JN589150.1, JN617194.1, JN680358.1, JN680360.1, JN696446.1, JN696453.1, JN900292.1, JQ011941.1, JQ033579.1, JQ041766.1, JQ062462.1, JQ062498.1, JQ218230.1, JQ230971.1, JQ230973.1, JQ230979.1, JQ283863.1, JQ388495.1, JQ388496.1, JQ388506.1, JQ388510.1, JQ392363.1, JQ392373.1, JQ392414.1, JQ392422.1, JQ392460.1, JQ392467.1, JQ405006.1, JQ424123.1, JQ669074.1, JQ669099.1, JQ669106.1, JQ669130.1, JQ669135.1, JQ669138.1, JQ776896.1, JQ792200.1, JQ895237.1, JQ898636.1, JX025227.1, JX073962.1, JX073965.1, JX078996.1, JX233505.1, JX233664.1, JX274203.1, JX274532.1, JX274538.1, JX464257.1, JX464294.1, JX467613.1, JX475146.1, JX576677.1, JX852437.1, JX867624.1, JX867643.1, KC156645.1, KC292629.1, KC455645.1, KC480352.1, KC535851.1, KC539599.1, KC575608.1, KC677958.1, KC677967.1, KC691712.1, KC691717.1, KC698935.1, KC812809.1, KC832358.1, KC861841.1, KC897915.1, KC897943.1, KC899568.1, KC922442.1, KC922448.1, KC952021.1, KC952713.1, KC958857.1, KC995016.1, KF022352.1, KF022626.1, KF022689.1, KF022704.1, KF022705.1, KF022719.1, KF137926.1, KF137936.1, KF150555.1, KF160912.1, KF163848.1, KF196320.1, KF201880.1, KF234635.1, KF265370.1, KF265388.1, KF270901.1, KF270902.1, KF301217.1, KF419387.1, KF447287.1, KF454227.1, KF454242.1, KF454259.1, KF454271.1, KF454296.1, KF454647.1, KF454681.1, KF482106.1, KF482108.1, KF493795.1, KF529544.1, KF544630.1, KF544886.1, KF547215.1, KF553460.1, KF704394.1, KF707563.1, KF713173.1, KF713204.1, KF718373.1, KF784875.1, KF806584.1, KF849103.1, KF850575.1, KF850588.1, KF850594.1, KF850608.1, KF850611.1, KF850613.1, KF861968.1, KF866381.1, KF873575.1, KF873765.1, KF897521.1, KF977441.1, KF983155.1, KF986733.1, KF993463.1, KF993513.1, KJ011903.1, KJ021874.1, KJ023678.1, KJ157634.1, KJ157635.1, KJ188998.1, KJ400904.1, KJ400936.1, KJ415356.1, KJ418133.1, KJ460085.1, KJ473882.1, KJ473895.1, KJ477049.1, KJ525046.1, KJ598893.1, KJ598933.1, KJ598938.1, KJ598940.1, KJ598941.1, KJ598943.1, KJ598945.1, KJ598947.1, KJ598985.1, KJ598998.1, KJ599003.1, KJ718098.1, KJ768883.1, KJ787175.1, KJ787202.1, KJ829446.1, KJ848154.1, KJ849660.1, KJ884130.1, KJ884178.1, KJ918495.1, KJ939168.1, KJ941156.1, KJ956490.1, KJ959254.1, KJ999384.1, KJ999385.1, KM010363.1, KM051437.1, KM051448.1, KM051450.1, KM051454.1, KM051455.1, KM051464.1, KM064889.1, KM073067.1, KM077298.1, KM113798.1, KM347931.1, KM484884.1, KM582605.1, KM875628.1, KM887371.1, KM887379.1, KM887393.1, KM887399.1, KM894999.1, KM999964.1, KP057065.1, KP098092.1, KP100268.1, KP120058.1, KP133672.1, KP159318.1, KP174260.1, KP231354.1, KP297495.1, KP297506.1, KP334194.1, KP406141.1, KP682404.1, KP682406.1, KP682411.1, KP682414.1, KP725332.1, KP738156.1, KP751380.1, KP828810.1, KP871508.1, KP871514.1, KP873242.1, KP873338.1, KP873356.1, KP875310.1, KP972321.1, KR005087.1, KR005088.1, KR005155.1, KR011991.1, KR082780.1, KR870346.1, KT179623.1, KT249827.1, KT249843.1, KT249881.1, L11578.1, L35881.1, LC003517.1,

LC027281.1, LC027919.1, LC076495.1, LC090002.1, LC090009.1, LM997566.1, LN610779.1, U24017.1, U27686.1, U30541.1, U30594.2, U56010.1, U63181.1, U63186.1, U74407.1, U78417.1, U78440.1, U88194.1, U88199.1, U90783.1, U90798.1, U90799.1, U90802.1, U96865.1, X63199.1, X98629.1, X98632.1, Z98279.1

References

1. Moorhouse-Gann, R. Ecological replacement as a restoration tool: Disentangling the impacts and interactions of Aldabra giant tortoises (*Aldabrachelys gigantea*) using DNA metabarcoding. PhD thesis (Cardiff University, 2017).
2. Murton, R., Westwood, N. & Isaacson, A. The feeding habits of the woodpigeon *Columba palumbus*, stock dove *C. oenas* and turtle dove *Streptopelia turtur*. *Ibis* **106,** 174–188 (1964).
3. Browne, S. & Aebischer, N. Habitat use, foraging ecology and diet of Turtle Doves *Streptopelia turtur* in Britain. *Ibis* **145,** 572–582 (2003).
4. Dunn, J., Morris, A. & Grice, P. Testing bespoke management of foraging habitat for European turtle doves *Streptopelia turtur*. *J. Nat. Conserv.* **25,** 23–34 (2015).
5. Bates, D. & Maechler, M. lme4: Linear mixed-effects models using S4 classes. (2009).
6. R Core Team. R: A language and environment for statistical computing. (2016).
7. Breheny, P. & Burchett, W. visreg: Visualization of Regression Models. R package version 2.4-1. https://CRAN.R-project.org/package=visreg. (2017).
8. Dunn, J. *et al.* The decline of the Turtle Dove: dietary associations with body condition and competition with other columbids analysed using next generation sequencing. *Mol. Ecol.* accepted
9. Redfern, C. & Clark, J. *Ringers' Manual*. (British Trust for Ornithology, 2001).
10. Dunn, J. *et al.* Non-cultured faecal and gastrointestinal seed samples fail to detect Trichomonad infection in clinically and sub-clinically infected columbid birds. *Conserv. Genet. Resour.* **8,** 97–99 (2016).
11. Brown, D. *et al.* Dietary competition between the alien Asian Musk Shrew (*Suncus murinus*) and a re-introduced population of Telfair's Skink (*Leiolopisma telfairii*). *Mol. Ecol.* **23,** 3695–3705 (2014).
12. Bolger, A., Lohse, M. & Usadel, B. Trimmomatic: A flexible read trimming tool for Illumina NGS data. *Bioinformatics* btu170 (2014).
13. Magoč, T. & Salzberg, S. FLASH: Fast length adjustment of short reads to improve genome assemblies. *Bioinformatics* **27,** 2957–2963 (2011).
14. Schloss, P. *et al.* Introducing mothur: Open-source, platform-independent, community-supported software for describing and comparing microbial communities. *Appl. Environ. Microbiol.* **75,** 7537–7541 (2009).
15. Edgar, R. Search and clustering orders of magnitude faster than BLAST. *Bioinformatics* **26,** 2460–2461 (2010).
16. Bengtsson-Palme, J. *et al.* ITSx: Improved software detection and extraction of ITS1 and ITS2 from ribosomal ITS sequences of fungi and other eukaryotes for use in environmental sequencing. *Methods Ecol. Evol.* **4,** 914–919 (2013).
17. Hawkins, J. *et al.* Using DNA metabarcoding to identify the floral composition of honey: A new tool for investigating honey bee

foraging preferences. *PLoS One* **10,** 1–20 (2015).
18. Kircher, M., Sawyer, S. & Meyer, M. Double indexing overcomes inaccuracies in multiplex sequencing on the Illumina platform. *Nucleic Acids Res.* **40,** (2012).
19. Schnell, I., Bohmann, K. & Gilbert, M. Tag jumps illuminated - reducing sequence-to-sample misidentifications in metabarcoding studies. *Mol. Ecol. Resour.* **15,** 1289–1303 (2015).
20. Altschul, S. *et al.* Gapped BLAST and PSI-BLAST: a new generation of protein database search programs. *Nucleic Acids Res.* **25,** 3389–3402 (1997).
21. Camacho, C. *et al.* BLAST+: architecture and applications. *BMC Bioinformatics* **10,** 421 (2009).
22. de Vere, N. *et al.* Using DNA metabarcoding to investigate honey bee foraging reveals limited flower use despite high floral availability. *Sci. Rep.* **7,** 42838 (2017).
